# Sensorimotor Underpinnings of Mathematical Imagination: Qualitative Analysis

**DOI:** 10.3389/fpsyg.2021.692602

**Published:** 2022-01-18

**Authors:** Gin McCollum

**Affiliations:** Fariborz Maseeh Department of Mathematics and Statistics, Portland State University, Portland, OR, United States

**Keywords:** embodied cognition, mental Imagery, mathematics education, graph, algebra, sensorimotor, vestibular

## Abstract

Many mathematicians have a rich internal world of mental imagery. Using elementary mathematical skills, this study probes the mathematical imagination's sensorimotor foundations. Mental imagery is perturbed using body position: having the head and vestibular system in different positions with respect to gravity. No two mathematicians described the same imagery. Eight out of 11 habitually visualize, one uses sensorimotor imagery, and two do not habitually used mental imagery. Imagery was both intentional and partly autonomous. For example, coordinate planes rotated, drifted, wobbled, or slid down from vertical to horizontal. Parabolae slid into place or, on one side, a parabola arm reached upward in gravity. The sensorimotor foundation of imagery was evidenced in several ways. The imagery was placed with respect to the body. Further, the imagery had a variety of relationships to the body, such as the body being the coordinate system or the coordinate system being placed in front of the eyes for easy viewing by the mind's eye. The mind's eye, mind's arm, and awareness almost always obeyed the geometry of the real eye and arm. The imagery and body behaved as a dyad, so that the imagery moved or placed itself for the convenience of the mind's eye or arm, which in turn moved to follow the imagery. With eyes closed, participants created a peripersonal imagery space, along with the peripersonal space of the unseen environment. Although mathematics is fundamentally abstract, imagery was sometimes concrete or used a concrete substrate or was placed to avoid being inside concrete objects, such as furniture. Mathematicians varied in the numbers of components of mental imagery and the ways they interacted. The autonomy of the imagery was sometimes of mathematical interest, suggesting that the interaction of imagery habits and autonomy can be a source of mathematical creativity.

## 1. Introduction

Learning to read graphs in algebra, students learn to recognize and draw coordinate axes, one right-left, one vertical. They learn to judge the relationship between a curve and coordinate axes (Vinner and Dreyfus, 1989; Tall, 1991, 1997). Recognizing coordinate axes and judging the relationship of a curve to axes require both perceptual and eye-movement skills, as does reading text (Rayner, 1977; Elterman et al., 1980; Sun et al., 1985). Drawing graphs is even more clearly cognition that is grounded in the body and sensorimotor systems (Varela et al., 1991; Seitz, 2000; Barsalou et al., 2003; Barsalou, 2008). Reading graphs requires the directionality characteristic of the vestibular system (Péruch et al., 2011; Bigelow and Agrawal, 2015). With practice, skills become engrained, so that a mathematician teaching algebra at the college level uses graph-reading skills and mental imagery strategies and skills, which they use as a creative space in thinking about their research (Sfard, 1994).

This study probes the connection between the mathematical imagination, visual or not, and its sensorimotor foundation. Skills of spatial imagination, along with individual strategies, have been studied over populations using non-mathematical tasks (Kozhevnikov et al., 2005; Hegarty et al., 2018). In particular, spatial as opposed to object imagery involves analyzing the relationships between components of the imagery (Kozhevnikov et al., 2005). The direct connection between mathematical cognition and its sensorimotor foundation is made with concrete objects in elementary mathematics (for example, Fuson et al., 1997; Lowrie et al., 2018) but typically not with mature mathematics. The study of mathematical cognition itself is not new (Poincaré, 1908; Hadamard, 1945) and has grown into an enormous literature including studies of external imagery (Sfard, 1994; Ochs et al., 1996; Greiffenhagen, 2014).

Outside of mathematics, the connection between mental imagery and its sensorimotor foundation has been made through imaging and lesion studies (Farah, 1988; Kosslyn et al., 1995, 1999, 2004; Binkofski et al., 2000; O'Craven and Kanwisher, 2000; Tranel et al., 2003; Slotnick et al., 2005). A more direct connection has been made by manipulating head position with respect to gravity, to demonstrate the vestibular influence on assigned imagery (Corballis et al., 1978; Marendaz et al., 1993; Gaunet and Berthoz, 2000; Mast et al., 2003, 2014). In separate groups of upright, right-ear-down, and supine observers, Mast et al. (2003) used a mixed task in which participants saw on a computer screen an image onto which they projected a mental image of a letter or number. Right-ear-down participants were significantly less accurate in combining computer and mental images into one composed whole. However, upright participants were significantly less accurate than supine or right-ear-down participants in inspecting parts of the image.

The focus of the present study is on the sensorimotor foundation of mathematical imagery, including visual, kinaesthetic, and somatosensory. The experiment perturbs mathematicians' imaginative skills using gravity. Each mathematician produced mental imagery according to their professional habits, but in different body positions, with the head and vestibular system in different positions with respect to gravity. The resulting perturbation revealed each mathematician's imagery strategies, how they shifted, and the semi-autonomy of the imagery.

## 2. Experimental Methods

Mathematicians were asked to perform a familiar task, graphing algebraic equations, but in body positions in which they may not usually work, positions that place the head in different positions with respect to gravity. They also performed three-dimensional tasks: wrapping a spiral around a cylinder or cone. A formative factor in the methods and data analysis has been how pressed the mathematicians are for time, primarily because of teaching commitments, making recruitment of participants difficult.

### 2.1. Participants

Eleven mathematicians (4 female) were recruited from four institutions and a wide variety of specialties, including algebra, topology, computation, and mathematics education. Eight were mathematics professors, from assistant to full professor, and 3 were senior graduate assistants who teach mathematics at the university level. Each signed a statement of informed consent. The procedures were approved by the Portland State University Institutional Review Board. Participants were assigned arbitrary numbers and are specified P1-P11. Also, they are assigned a nickname for ease of reading, for example P8 Straight-ahead.

### 2.2. Tasks

Participants were asked to produce mental imagery with closed eyes:

- Task 0: Imagine a coordinate plane
- Task 1: Graph *y* = *x*^2^
- Task 2: Graph *x* = *y*^2^
- Task 3: Graph *y* = 1/*x*
- Task 4: Graph *y* = *x*(*x*^2^ − 1)
- Task 5: Imagine a cylinder and wrap a spiral around it
- Task 6: Imagine a cone and wrap a spiral around it

These items are not rote images, but mathematical structures that can be instantiated in many ways in imagery.

The entire series was introduced by a request to arrange a place and time where the participant could lie down undisturbed, in keeping with the typical solitary nature of mathematical work. Each task was introduced by a short paragraph asking questions intended to evoke further description, which it did. In Tasks 3,4, participants were asked to follow the graph from negative to positive. Full instructions are found in the Supplementary Materials.

All participants except P1, P9, and P10 performed the whole series of tasks in one session. P1 and P10 followed the written instructions and did the study in two sessions in 1 day, P1 following T0-T3 in the morning and the rest in the late afternoon and P10 performing T0 in the late afternoon and the rest in the late evening. P9, in person, was tired after T3; we resumed the experiment the following week.

**Figure 1:**
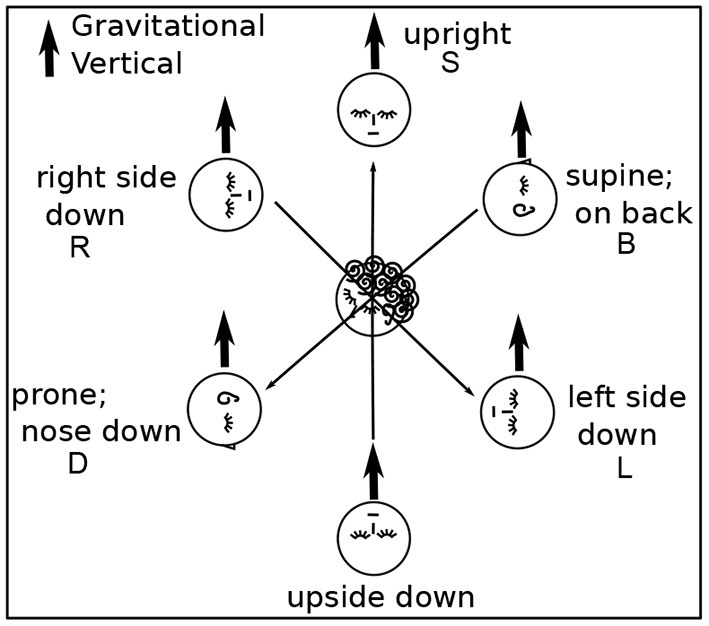
Head Positions with Respect to Gravity. Gravity can be aligned with three axes through the head, in two directions along each axis. Participants assumed each position except upside down. Positions are denoted B, R, L, D, and S.

**Figure 2:**
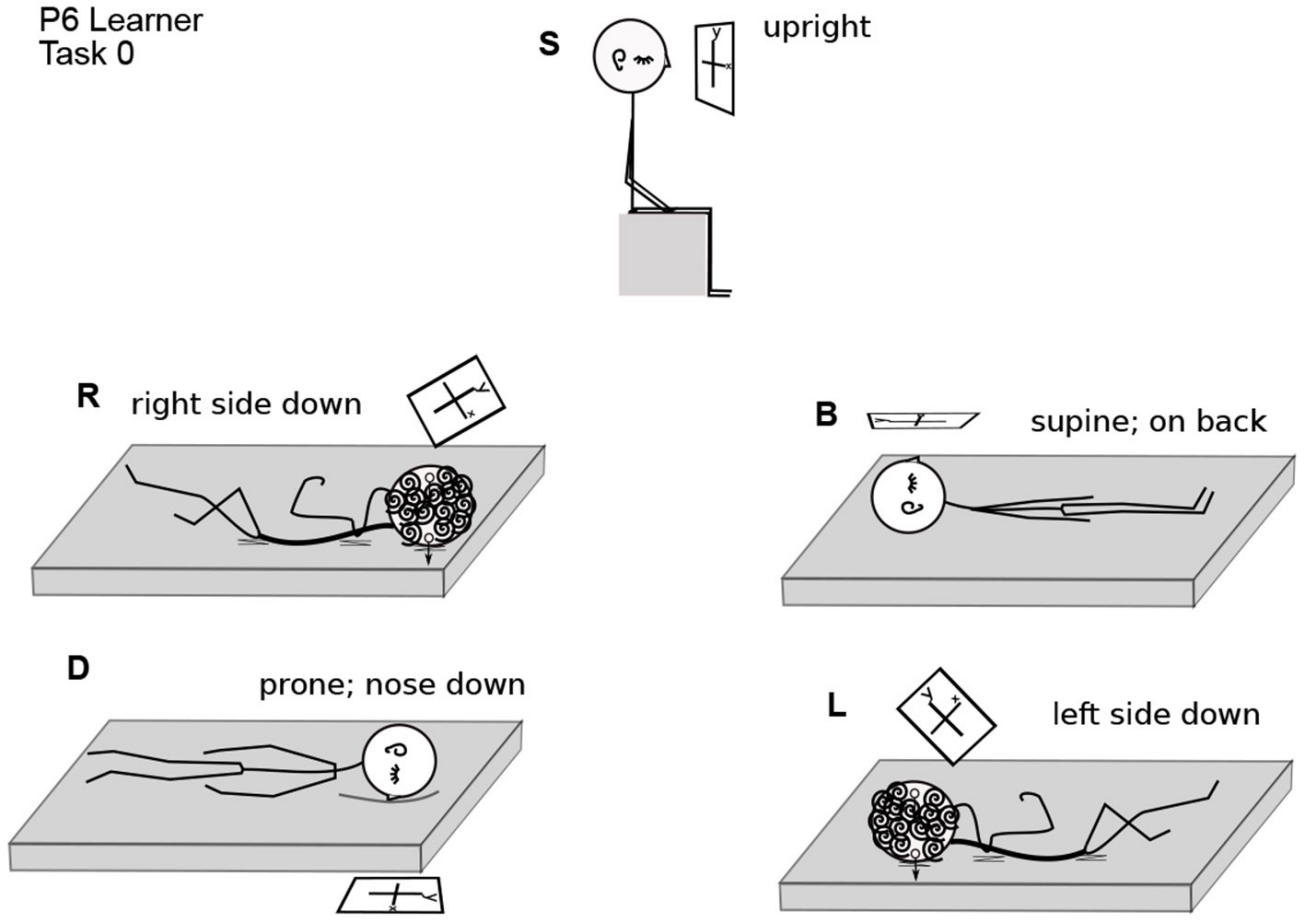
Participant 6 Performing Task 0. Body positions are arranged as in Figure 1. The coordinate plane is drawn in a rectangle, as if on a piece of paper, to make its orientation clearer. In position S, P6 is upright in gravity. The coordinate plane imagery is in front of the face, gravitationally vertical and parallel to the frontal (also called lateral) plane of the body. In R, the right ear is down. The coordinate plane imagery is in front of the face, with the *y*-axis on a diagonal between body-parallel and gravitationally vertical. In B, the nose is pointing up in gravity. The coordinate plane is in a horizontal plane with respect to gravity, in front of the face, with the *y*-axis parallel to the body axis. In D, the nose is down. The coordinate plane is horizontal, parallel to the floor, and the *y*-axis is body-parallel. In L, the left ear is down. The coordinate plane is in front of the face, with the *y*-axis on a diagonal between body-parallel and gravitationally vertical.

Participants were asked to perform each task in various body positions: lying on their backs (B), right sides (R), left sides (L), face down (D), or sitting (S) (Figures 1, 2). Some participants assumed the body positions in the sequence mentioned; some varied the sequence from task to task, as specified when relevant in the Results, for example, in **Figure 8**. A trial is specified by participant number Px, task number Tx, and body position, for example P3T4L.

### 2.3. Recording Data

Eight of the 11 participants performed the experiment at their own convenience. Of these eight, P6 drew diagrams and made notes around them, P7 made an audio recording as the imagery developed, and the other six (P1, P2, P3, P5, P8, and P10) wrote brief but rather expressive notes immediately after each body position. Three (P4, P9, and P11) requested in-person experimental sessions, and their descriptions, including gestures, were recorded in notes by the experimenter. Although the same task instructions were read in person, the verbal and gestural descriptions led to more freedom being taken with the instructions and to some illuminating side comments that are reported in the Results.

Perhaps the most important factor in the recording of the data was that the participants, overall, were surprised and amused at the imagery. Especially the verbal descriptions expressed the deviations of the mental imagery from textbook drawings and participants' everyday use of imagery in their work, in their usual body positions.

### 2.4. Data Analysis

The overwhelming variety of responses was gradually reduced by comparing participants' responses to identify commonalities and differences. This is the method of constant comparison. As more specific comparative questions arose, the original data were queried again for further detail.

#### 2.4.1. Drawing

Only P6 supplied drawings. All other imagery described by participants was drawn by the experimenter from verbal and gestural descriptions, as in Figures 2–4, 6–11. The drawing process forced a deeper querying of each description.

**Figure 3:**
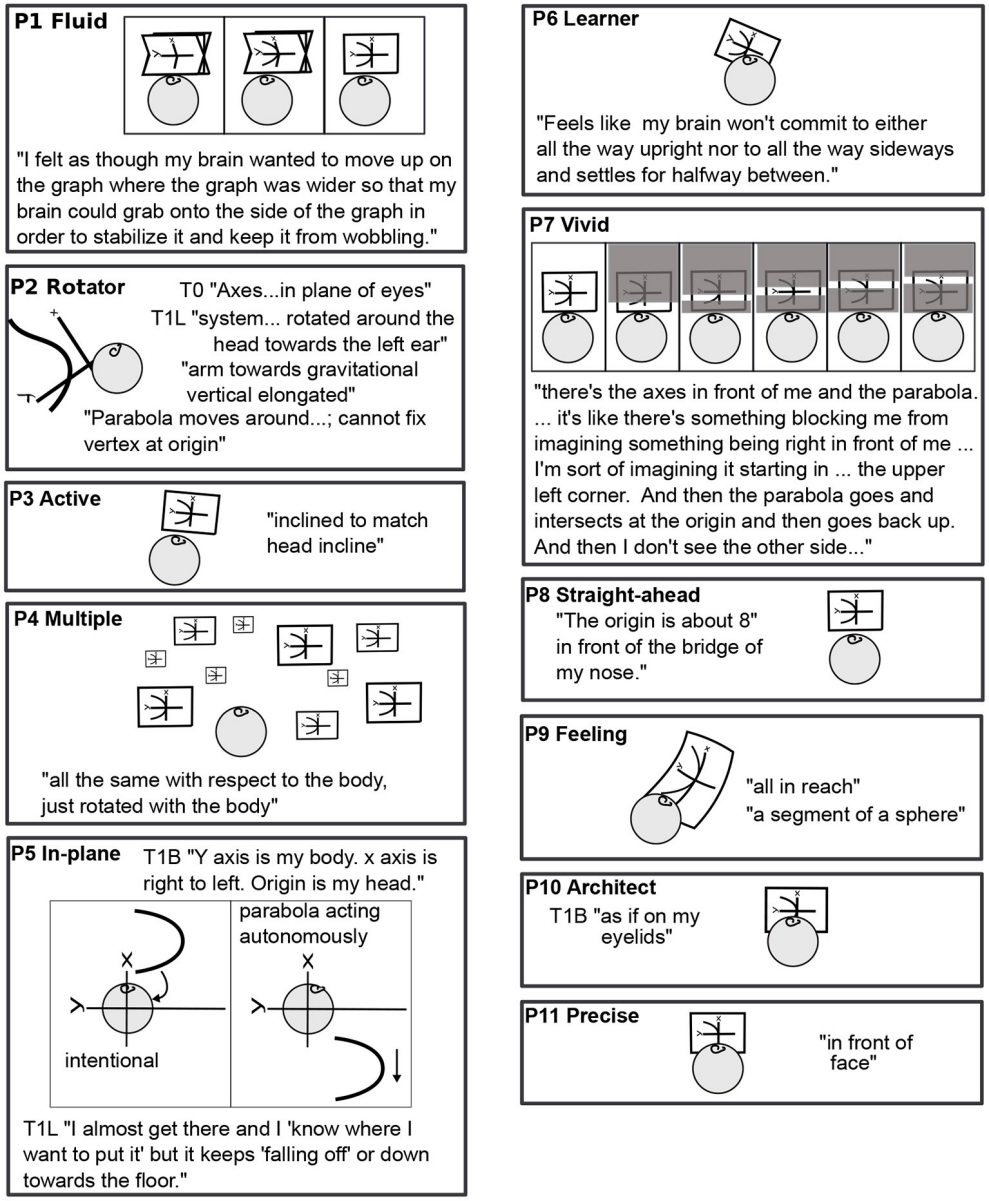
Task 1 L drawn for all participants: Variety. Sequential drawings (P1, P5, and P7) read left to right, like a comic strip. The right ear up shows that the participant is lying on the left side. P2's head is rotated in backward pitch to show the position of the plane (see Figure 4, lower right corner, for pitch, roll, and yaw rotations). Quotes are taken from other body positions (P5, P10) when it is clear from context that they apply to T1L.

**Figure 4:**
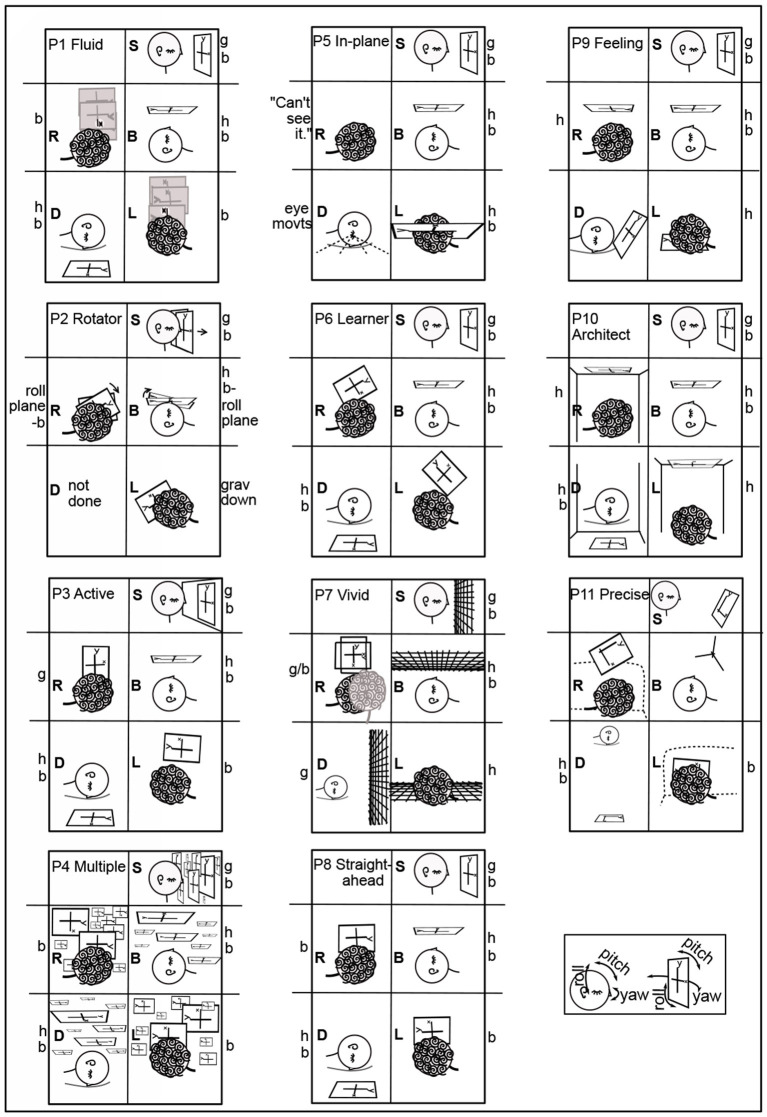
All participants' imagery in T0. From verbal descriptions and gestures, including context, the orientation and placement of the coordinate plane in Task 0 is drawn for each participant and each body position (Figures 1, 2). The box for each participant roughly follows Figures 1, 2 head placement. Coordinate planes are drawn with frames for clarity, even though the appearance of coordinate planes varied among participants. A large y is shown at the top of the *y*-axis for clarity. The head in each drawing indicates its direction, usually by the eyes and/or an ear. The orientation of the head in each drawing is chosen to best depict the imagery and its placement, not necessarily the participant's position, which is indicated by letter to the left of each drawing. Particular plane orientations are marked at the side of the drawing: b - plane and *y*-axis body-parallel; g - *y*-axis gravitationally vertical and plane parallel to frontal plane; h - plane horizontal (orthogonal to gravity). P1's plane moved fluidly in R, L, so that the area of interest was in front of the eyes (3.2.4). P2's coordinate plane was in the plane of the eyes. In S, P3's coordinate plane was on an imaginary wall. In L, P5 was in the plane “as it was on my back” (3.2.2). In D, P5 said “My eye movements assist in attempting to orient myself;” P5 used eye movements in an unsuccessful attempt to create a plane like that in L. P7's coordinate planes were typically grids, rather than having axes. For P7R, a second head indicates a perspective shift (3.2.1). In D, P9's coordinate plane is rotated in pitch above the head. In R, L, P10's coordinate plane is on the ceiling, and in D, on the floor. In L, the *y*-axis is along the wall-ceiling corner. P11 prefers visualizing in three dimensions, aa illustrated in B. This preference led to the coordinate plane in S being tilted in pitch and roll. In R, P11's coordinate plane is raised to avoid being in the couch back, shown as a dashed line in R,L. Roll, pitch, and yaw rotational directions are illustrated in the lower right corner for both the head and the imagery.

**Figure 5:**
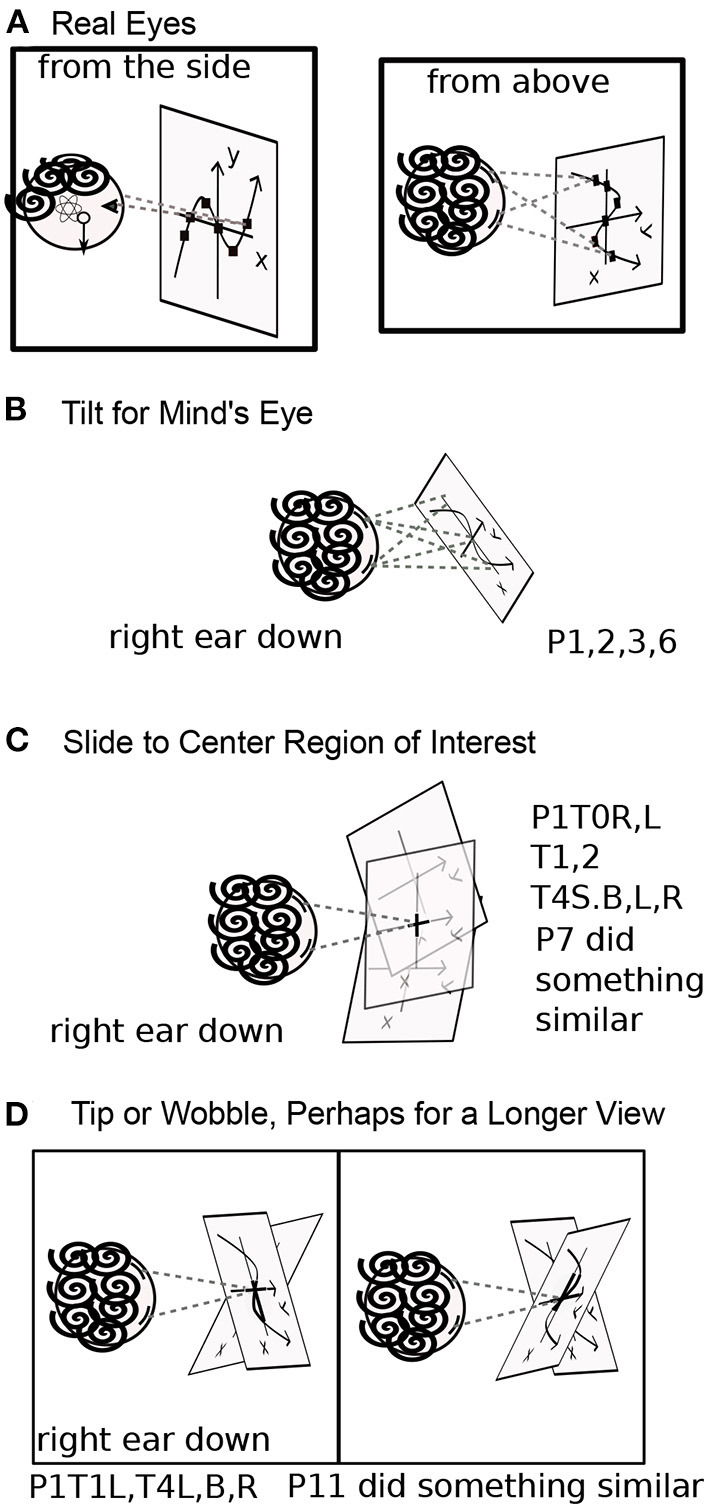
Convenience for the Mind's eye. **(A)** Real eyes reading a graph with eye movements mainly in the plane of the eyes themselves, horizontal to the body. **(B)** Participant in R (eyes closed) with the graph tilted on a diagonal between body-vertical and gravitational-vertical. Dashed lines show the mind's eye reading the imagery graph. P1,2,3,6 used such a tilt. **(C)** P1 with sliding graph. Dashed lines show that the mind's eye looks forward and does not move. Instead, the graph slides so that the region of interest is straight ahead. Only the focal area of the mind's eye is clear. P7's graphs also slid, with parts visible and parts obscured, as in T1L (Figure 3). **(D)** P1 with graph tipping or wobbling about the *y*-axis, in yaw. As in C, the eyes look straight ahead. The two frames show the two extreme tipped positions. Only the center is clear in each, and each gives a view along the *x*-axis. P11's imagery also rotated or tipped in yaw for a better view in R (T2-4, T5,6).

**Figure 6:**
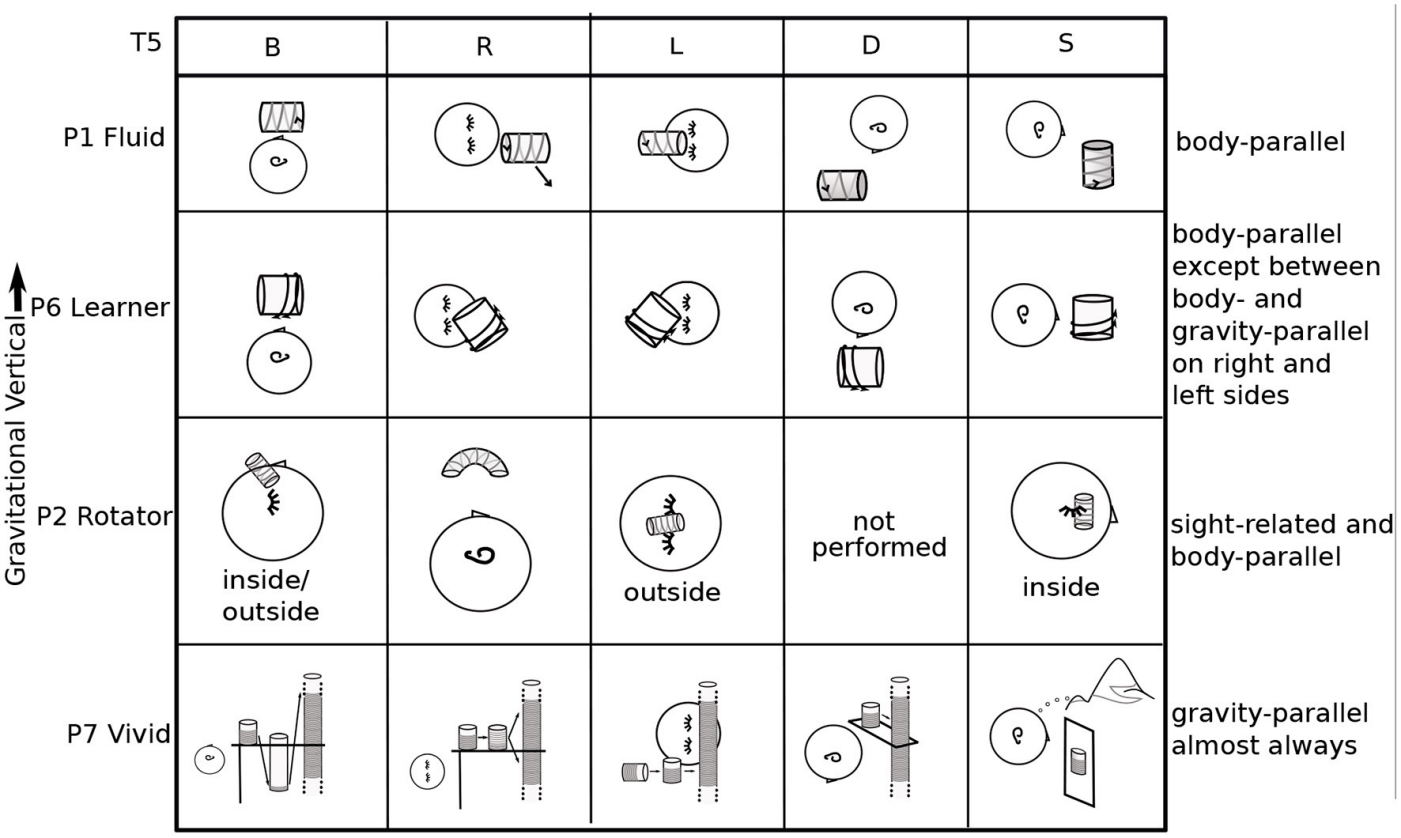
Variety of placement and orientation patterns in T5. The columns are the five body positions. The rows are four participants (P1,6,2,7) who exemplify body, gravity, and architectural references and their variations and mixtures. P1's imagery is oriented parallel to the body axis. P6's is also, except in R,L, when it is diagonal, between body-parallel and gravity-upright. P2 sometimes positioned imagery inside the body. In R, the cylinder is curved, with the top and bottom “visible” to the mind's eye. P7's cylinders are almost always oriented upright in gravity, with an exception in L.).

**Figure 7:**
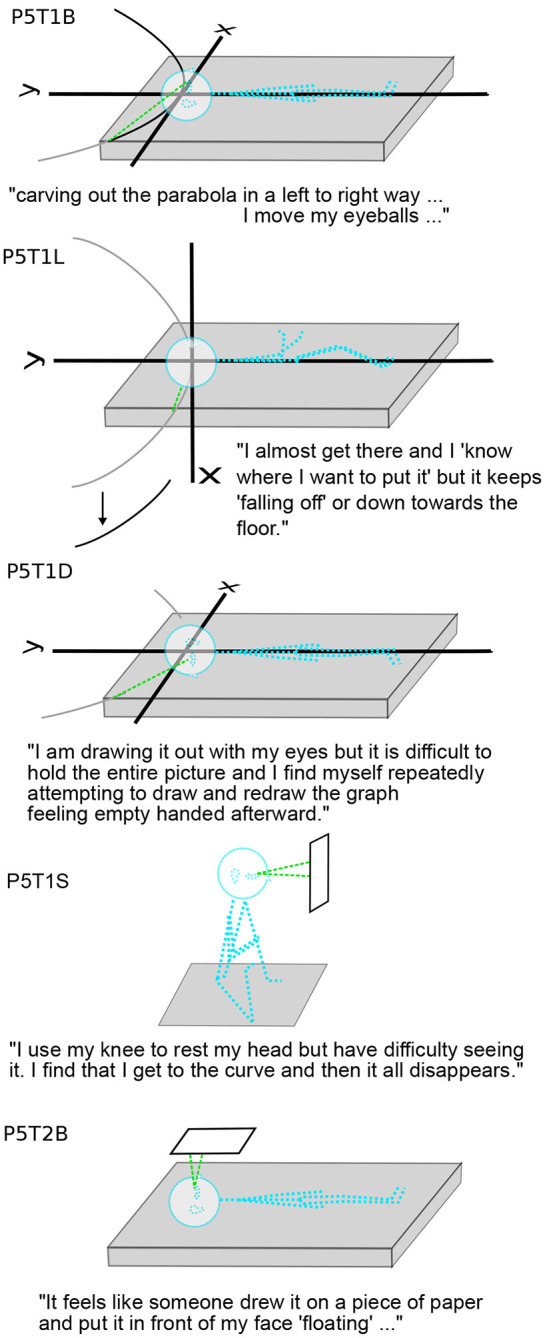
Shift from body-as-coordinates to in front of face. P5T1B, L, D show P5's struggles to draw a parabola with eye movements on a coordinate system that is the body. The gray parabola is the intended parabola that is achieved in B but not in L or D. P5 makes the dramatic transition to a coordinate system parallel to but outside the body, along with looking at an already-drawn graph.).

**Figure 8:**
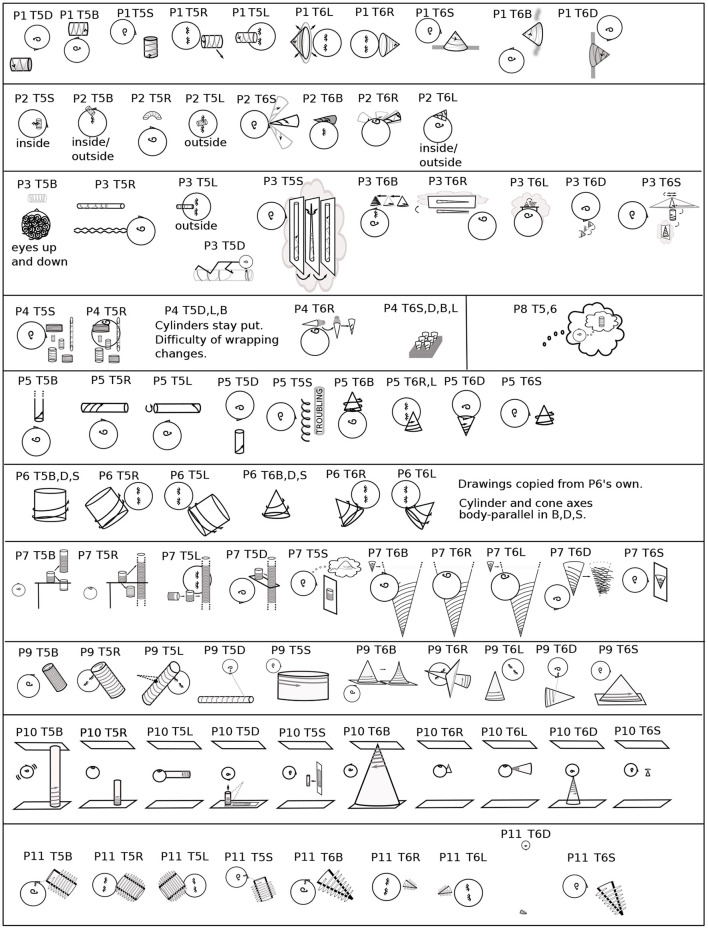
Tasks 5, 6, Placement and orientation. Sketches are in sequence of performance. Each sketch is labeled with participant, task, and body position. For head position, note the orientation of the ear and placement of nose and closed eyes. Drawn head position may vary from body position to allow imagery to be seen. Drawings are from verbal and gestural descriptions except for P6, who provided drawings. The multiplicity of P4's imagery is given as a possible example. (P8 is next to P4, to save space).

**Figure 9:**
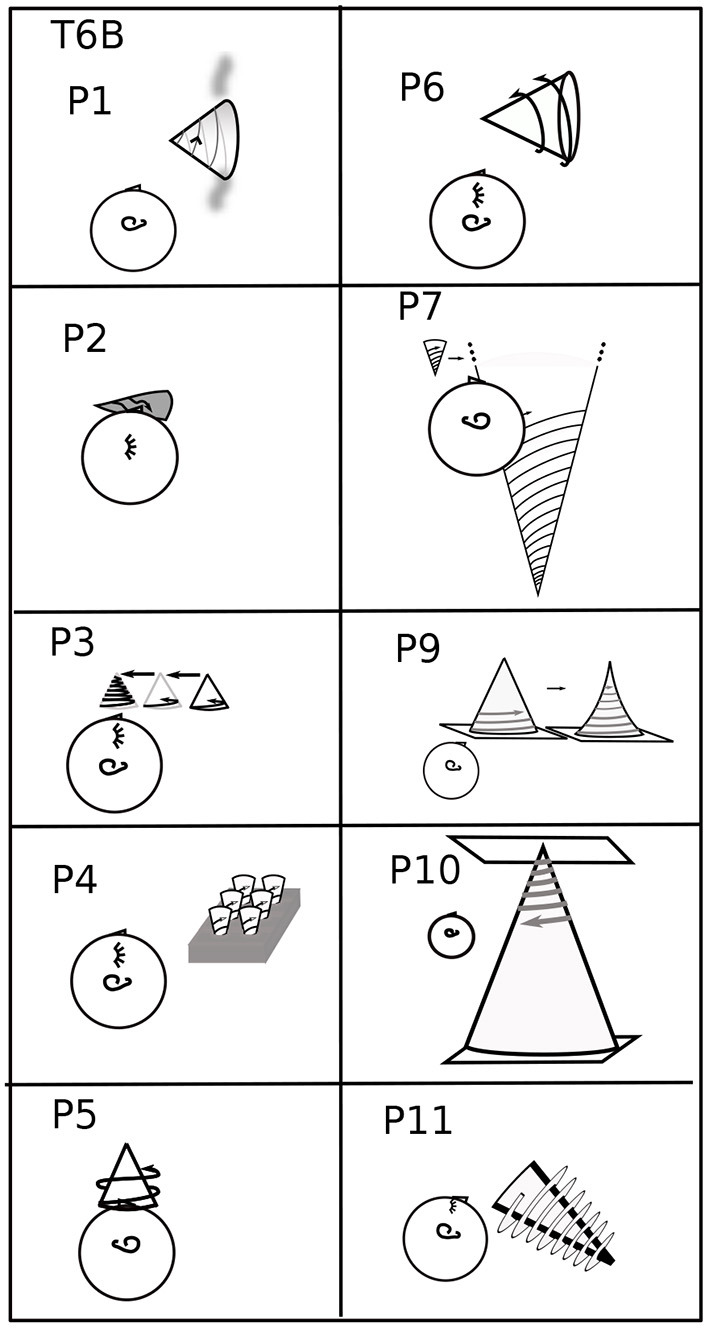
Perspective problem in T6B. The surface on which P1's cone is standing is “wavy, moving, and bluish.” P11's spirals are flat, like scarves sticking out from the cone. P8 is omitted because the cone and spiral were denoted. Floor and ceiling are depicted for P10. P4's cones are ice-cream cones in a cone holder. Perspective is problematic for P4, P7, P9, and P10, although P9 was the only one who mentioned it explicitly.

**Figure 10:**
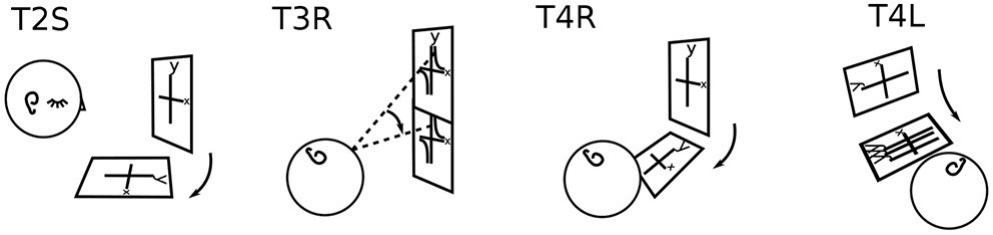
P3's imagery moving with eye movements. Behind closed eyelids, P3's eye movements led the imagery from vertical to horizontal in three cases and to a lower vertical in the fourth.

**Figure 11:**
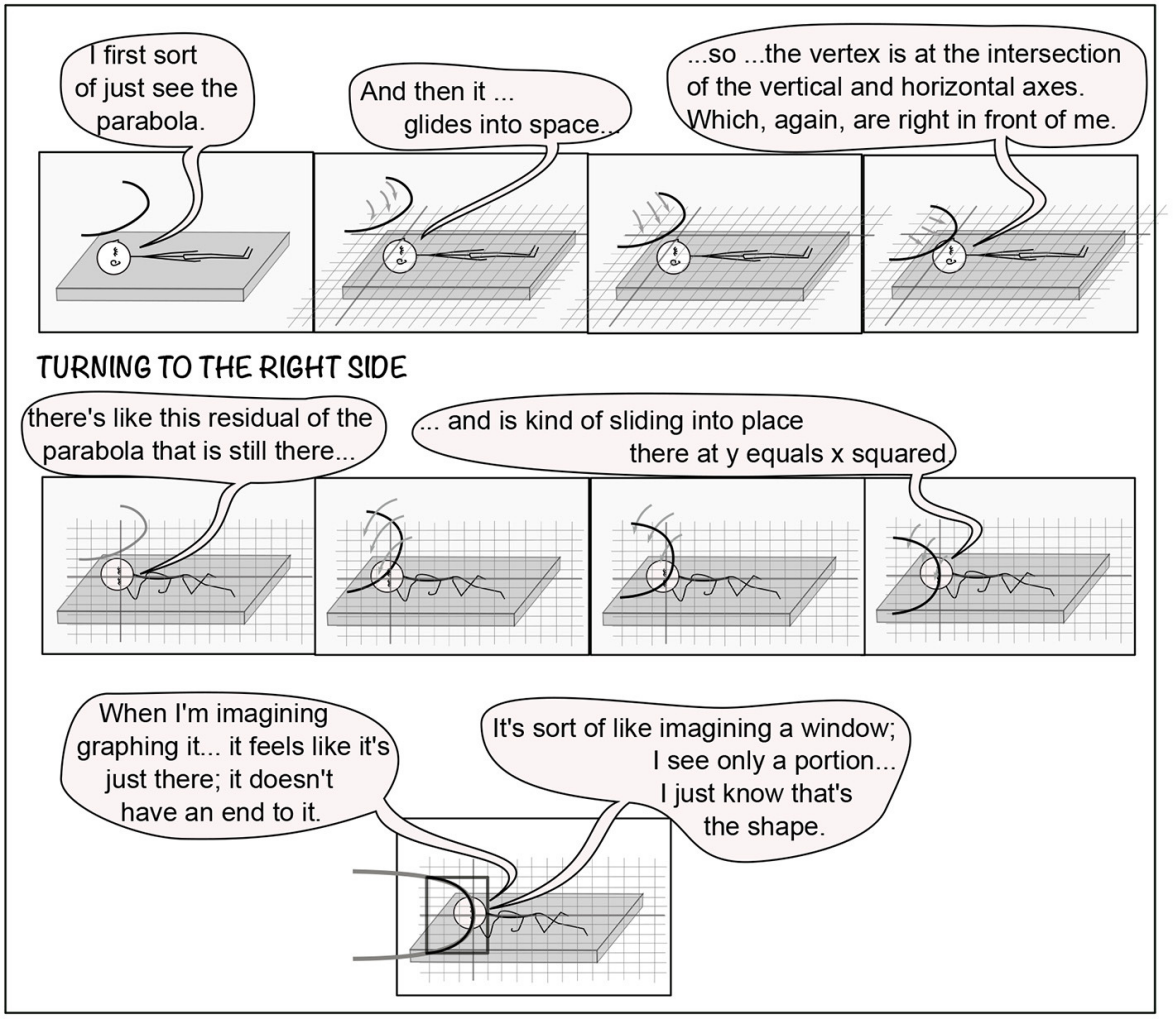
Components or layers in P7T1B,R. Top row, left to right: P7's sequence of visualizations in T1B, along with P7's verbal description. Drawings are from P7's verbal account, including the context from previous tasks. P7 typically graphed on a grid, rather than axes. The second row begins with P7 turning to the right side, with the parabola remaining in place. The parabola rotates in yaw and slides into place on the grid. The third row shows P7's further description.

#### 2.4.2. Assessing Sensory and Sensorimotor Relationships

The *y*-axis is typically “up” on a graph, but where is up? In imagination, mathematicians could choose any direction as up. Relationships such as the direction of up were assessed from the mathematicians' descriptions of the imagery. Mathematicians positioned imagery in peripersonal space and described axes as parallel to the gravitational upward direction, up with respect to the body, or a mixture. They also described directions and positions with respect to architectural features, such as floors, walls, and ceilings.

Participants described geometrical relationships, which allowed inference of sensory and sensorimotor relationships. For example, visual imagery was usually placed where it could be seen with the eyes, had they been open. There was considerable explicit variation, for example, following a spiral with “awareness” when it was behind a cylinder or cone and could not be “seen” with the mind's eye. Attention or awareness is often studied in connection with eye movements and intentional following of images (Corbetta et al., 1998; Buschman and Miller, 2007; Zhao et al., 2012; Johansson, 2013). Thus, descriptions such as “hard to traverse curve L to R across *y*-axis in my mind” (P6T3B) were interpreted as movement of attention or awareness.

## 3. Results

The data consist of mathematicians' descriptions of imagery experiences as they performed the tasks, along with comments (Figure 2). For example, P6 Learner commented of lying on the left side (L): “Hard to imagine anyone liking to work in this position.” Evidently P6 uses imagery as a creative space (Sfard, 1994). Following a subsection on the variety of imagery (3.1), sensorimotor relationships are presented (3.2), and then some of the properties of mathematical imagination (3.3–5).

### 3.1. Individuality

No two mathematicians had the same experiences of imagery overall. For example, Figure 3 lists all participants' experiences in Task 1, graphing the parabola *y* = *x*^2^, when lying on the left side (L). Each experience is part of a longer story, particular to each individual.

As shown in Figure 3, P1 Fluid's coordinate plane wobbles about the *y* axis, which is parallel to the body axis, gradually stabilizing over 5 min, after the parabola is added. P2 Rotator's plane is rotated around the head toward the left, the arm of the parabola reaches upward in gravity, and the parabola moves in the plane, its vertex not fixed at the origin.

The straightforward, body-parallel view of P3 Active, P8 Straight-ahead, P10 Architect, and P11 Precise are similar, at different distances. P10's and P2 Rotator's are “in the plane of the eyes.”

P5 In-plane's parabola falls toward the floor. P5 and P8 Straight-ahead do not habitually use mental imagery, visual or otherwise. Although the lack of visualization surprises many mathematicians, it is known in the literature. For example, John von Neumann apparently did not habitually visualize (Ulam, 1958).

P6 Learner's plane is halfway between gravitational and body upright. Something blocks full view of P7 Vivid's parabola, but allows a scan along it.

P9 Feeling does not use visual but rather somatosensory imagery. P9's plane is a segment of a sphere, all in reach of the mind's arm. Of the 11 mathematicians, 8 used visual imagery, 1 somatosensory imagery, and 2 used no mental imagery habitually. When mental imagery was used, it was primarily of the spatial rather than object type (Kozhevnikov et al., 2005), whether it was visual or somatosensory.

P4 Multiple pointed out that coordinates are abstract and have no position. P4 visualized them as “a bunch, all floating around. There is an equivalence class. Some are smaller, some bigger.” The instruction was to “imagine the axes in a rectangular coordinate plane.” The article “a” can be understood as “one particular” or “the generic.” One would have a similar choice in imagining a dog. One could visualize a particular dog. To imagine the generic dog, there are many alternatives, including visualizing a dog continually morphing in shape, size, color, and breed. Mathematically, the difference between particular and generic is significant. For example, “consider a group” could lead to considering the symmetry group of an equilateral triangle. Typically, “consider a group” means to consider the generic group as given by the definition. To visualize origins everywhere was to make generic thinking visually explicit. Other participants made the same point in different ways. P1 Fluid said the instructions felt a bit like “Imagine happiness. Where is it in space?” P9 Feeling couldn't feel the axes without engaging with them: “It's like sitting down at the piano, so I could play, but haven't yet. Like maybe you don't know what you're going to play.” For cones in Task 5, instead of imaging an equivalence class, P4 Multiple waited: “The generic cone isn't complete. I'm waiting for further instructions. It's like facing a pottery wheel. There are a hundred possible cones.” All imagery makes choices or compromises between the particular and generic, which are mathematically significant.

### 3.2. Sensorimotor Foundations of Mathematical Imagery

Mental imagery is produced by neural centers overlapping with those for external perception and action (Farah, 1988; Kosslyn et al., 1995, 1999, 2004; Binkofski et al., 2000; O'Craven and Kanwisher, 2000; Tranel et al., 2003; Slotnick et al., 2005; Lotze and Halsband, 2006). Manipulation of the vestibular sense provides a more direct demonstration of the sensorimotor underpinnings of imagery (Corballis et al., 1978; Marendaz et al., 1993; Gaunet and Berthoz, 2000; Mast et al., 2003, 2014). This section presents direct evidence of this sensorimotor foundation from within mathematical imagery.

#### 3.2.1. Coordinate Plane Orientation

Imagery was mostly placed according to the body and/or gravity, typically autonomously, that is, without or against the intention of the imager. For example, although P6 Learner's coordinate plane in T0B was body-parallel, it turned diagonal in R,L (Figures 1, 2), and P6 remarked, “There's tension here.” Eyes closed, participants were both placing an image as in placing a picture on a wall and looking at the imagery as one would look at a blackboard or watch a movie. Placing the coordinate plane (Task 0) is a foundation for using mathematical imagery, typically for thinking or for discussing a concept with a colleague. Figure 4 shows orientations and placements of the coordinate plane for all participants.

In 44 cases, the coordinate plane is gravitationally vertical (g), horizontal with respect to gravity (h), or body-parallel (b) (Figure 4). Gravitationally vertical and horizontal are conventional, as in blackboards and tabletops. Biologically, they are important sensorimotor directions for balancing and moving in gravitation.

In S, body axis and gravity are parallel. Almost all participants' planes were on or in front of the face, body-parallel and gravitationally upright (Figure 4). The exception is P11 Precise, whose plane was tilted, a habit useful in visualizing in three dimensions. In B, where the body axis is orthogonal to gravity, almost all planes were body-parallel and horizontal (Figure 4). P2's y-axes were body-parallel but drifted right in S and rolled clockwise in B. (Pitch, roll, and yaw rotational directions are indicated in the lower right of Figure 4).

In D, R, and L, also with body axis orthogonal to gravity, placement was more variable. The coordinate plane was placed in front of the face with the *y*-axis body-parallel by less participants: D - 6; R - 3 (and partly by P7); L - 5.

There was a major difference in the ways placement and orientation varied in D vs. R,L. In D, the *y*-axis was always in the sagittal plane (mid-plane of the body). P9 Feeling's plane was rotated in pitch above the head, not one of the three standard positions (gravitationally vertical, body-parallel, or horizontal). P7 Vivid's plane was rotated farther in pitch to gravitationally vertical. P4 Multiple's plane was behind the head and horizontal.

Planes were horizontal twice in R and four times in L (Figure 4). In R with the plane on the ceiling, P10 commented: “I feel a little disoriented in this position.” In L, when the coordinate plane was floor-parallel, P7 commented “I can feel the tension of looking at it from an unusual perspective.”

The plane was in a rolled position for five participants (P2, P3, P6, P7, and P11) in R and L and never in D. In R, P7 Vivid used the dramatic compromise of “switching back and forth” between gravitationally vertical and horizontal or body-parallel “sort of like what happens in an optical illusion” (Figure 4). However, “...the rectangle doesn't move, but I imagined my perspective changing ...” (Other examples of changing perspective to a position out of the body were P1T5B to better “see” the spiral and P4T3D imagining turning over to manipulate the graph above in 3.2.2.)

One reason a rolled plane may be preferred in R and L is that when the head tilts, the eyes tort (rotate about the pupillary axis) (Liebowitz et al., 1986; Schworm et al., 2002; Pansell et al., 2003; Ferrè et al., 2015). Eye torsion changes the balance of eye muscles in eye movements. Consequently, torted eye movements are less accurate (Wood et al., 1998). Reading graphs involves habitual eye movements, as does reading text (Rayner, 1977; Elterman et al., 1980; Sun et al., 1985) (Figure 5). Although reading is possible with the head completely to one side, in R,L, reading may be more comfortable at a diagonal.

These differences between D vs. R and L carried through in the rest of the coordinate plane tasks (T0-4) (Table 1). Coordinate planes were never rolled in D except in the arguable case when P4 Multiple left the equivalence class of planes body-parallel in L when turning to D (3.2.3). Instead, the coordinate planes were pitched in the sagittal plane in 10 cases in D. In contrast, coordinate planes were rolled 17.5 times in R and 11 times in L. They were never pitched.

**Table 1:** Orientation and distance.

**Participant**	**Orientation**	**Distance**
P1 Fluid	body-parallel, sometimes with slide, wobble, or tilt	paper distance
P2 Rotator	body-parallel with various rolls and drifts; yaw in T1L	T0-4 on eyelids; T5,6 just in front of eyes, on nose, and inside head
P3 Active	Sbg,Db; Bb except gravitational vertical in T6; Lb except tilted or gravitational vertical in T5,6; R b in T5,6; R diagonal in T2; otherwise R g	paper distance except T3R on far wall
P4 Multiple	body-parallel	paper distance; multiple
P5 In-plane nv	body-parallel except gravitational vertical in T6	T0,1B,R,L,D identity; T0,1S,T2,3,5,T6R,L,S paper distance; T6B,D on face
P6 Learner	body-parallel in S,B,D; diagonal toward gravitational vertical in R,L	paper distance
P7 Vivid	T0 Figure 4; T1-4 body-parallel; T5,6 almost all gravitational vertical	paper distance; T0,3,5,6D, T5L on wall; T5B,R on kitchen counter
P8 Straight-ahead nv	body-parallel	paper distance
P9 Feeling nv	body-parallel in S,D,T0B, T5R,L; tilted toward gravitational vertical in T1-5B; gravitational vertical in T6B,L; T0R,L horizontal; T1-4R,L spherical and with yaw; T6R diagonal with yaw	arm's distance; T4,5D more than arm's distance; projective when using eye movements
P10 Architect	S,B,D b, T0-4; R,L T0 horizontal; Lb T1-3; Lg T4; Rg T1-4; T5,6 gravitational vertical except T5D two perspectives 3.5	T0B, T1B,R,L on eyelids T0R,L on ceiling; T0-6D on floor: T0,1S, T2-4B,R,S, T2,4L, T5L,S, T6R.L,S paper distance; T3L on wall; T5 B,R,D, T6B rising from ground
P11 Precise	body-parallel except R diagonal and tilts in S,B in T0,1 and in T5,6 with roll to see top	paper distance; D 4-6'

*Evidence of a relationship to the body and the sensorimotor system was widespread, as shown in this table. “Orientation” summarizes for all tasks the information covered in Figure 4 for T0. “Distance” includes information about references, including placement for visual convenience or on architectural features. The description “paper distance” includes various visually-comfortable distances, from a few inches to arm's distance. “Identity” means that the body is the coordinate system*.

#### 3.2.2. Relationship to Body and Architecture

In addition to orientation, imagery is related to the body in something like attachment (Figure 6 and Tables 1, 2). This section presents the variety in that relationship.

**Table 2:** Relationship to body.

**Participant**	**Following and other manipulation**	**Imagery adjusts to body**
P1 Fluid	T1L brain wanted to grab graph; T3-6 awareness followed; T5R kept putting cylinder back in front; T6L stopped cone widening	T0-4 plane shifts to gaze direction; T0R,L T2L fluid position; T1L, T4B,R,L yaw wobble (Figure 5)
P2 Rotator	can see when cone or cylinder is transparent or positioned to see	T6S cone rotates about vertex, which is at bridge of nose (Figure 8)
P3 Active	T1,2 and 3,4S graph drawn; T4R bring plane back up; T5 follow autonomous spiral; T5B follow with eyes; T5S force cone back open to cylinder; T6 draw spiral: T6R turn 3D cone into chalk drawing: T6D turn cone into earth-boring drill	T4B,R,L,D graph emerges; T2S, T3R, T4R,L plane moves down with eyes (Figure 10)
P4 Multiple	will imagery to move with body: T3B construct graph; T3R follow with eyes; T3D bring one image in front to manipulate; T5,6 wrap cylinders and cones	T3,5,6 manipulate one and the rest follow
P5 In-plane nv	T1 carving, putting, drawing; T3B,D jump up and carve out; T5S actively wrap	T2 put in front of face
P6 Learner	T3-6 follow with awareness	
P7 Vivid	T4 graphing and graph appearing; T5 imagining can and spiral becomes autonomous; T6 wrapping spiral, then spiral becomes autonomous	imagery appears; T1,3R parabola rotates to follow P7
P8 Straight-ahead nv	T1 drew parabola; T2 pictured graph as if drawing it; T3,4 imagined graph all at once	
P9 Feeling nv	T1D can't get to it; T3B,R,L,S trace with head; T3D,T4R.S,T5D, T6D follow with eye movements; T4,5L follow with virtual hand movements; T4D attention moved along	graphs just there except T4B
P10 Architect	following autonomous graph; T5D force into 3D and cylinder rises	T5D force into 3D and cylinder rises
P11 Precise	T3,4 self black dot moving along; T5,6 mind's eye moves along	T5,6 spiral is scarf emerging as eye moves along

*Evidence of a relationship to the body and the sensorimotor system included not only the position and orientation to the face (Table 1) but further a variety of actions. “Following and Other Manipulation” shows the distribution of interactions described in the text. “Imagery Adjusts to Body” complements the more active manipulations, displaying the cooperative nature of body and imagery placement and movement. Especially P3,4,7,10, and 11 displayed imager/imagery cooperation*.

P5 In-plane reported the closest relationship between the body and the coordinate axes: identity. P5 does not habitually use mental imagery in mathematics, so these tasks were a special effort, as was verbalizing the experience. The coordinate plane in T0B was horizontal. Then in L: “I feel like I am in the plane as it was on my back and I can't find myself in it.” P5 started T1 with the *y*-axis being the body axis, the origin the head, and the *x*-axis right to left. In Task 1, P5 used eye movements to draw parabolae (Figure 7). Eye-movement drawing resulted in a stable parabola in B. In L, the drawn parabola or parts of it kept falling toward the floor, downward in gravity. Although gravity is a major reference for imagery overall—for example resulting in P6's tilted graphs in L and R (Figure 6)—it was more unusual for particular parts of imagery to move or be distorted by gravity. Other examples occurred in P2T1L (“arm toward gravitational vertical elongated”), P4T6R (icecream falling off cone), and P11T1R (arm of parabola rising in gravity). In D, as in L, P5 struggles to draw a parabola, but it never stabilizes (Figure 7). In S the coordinate plane is outside the body, and apparently P5 is using eye movements to draw the parabola, but it disappears. In T2, graphing *x* = *y*^2^, the parabola opens toward the positive *x*-axis. In T2B, without P5's effort or intention, the visualization is dramatically easier. The coordinate plane in T2 is not only separate from the body but also the graph is pre-drawn. This change in production of imagery occurred for many participants, but not so dramatically. For example, P3 drew parabolae, but later graphs appeared, all outside the body.

P2 Rotator reported the next closest relationship between body and imagery: the cylinder was inside the head in T5S (Figure 6). In T5B, it was partly inside and partly outside, as was the cone in T6L, but it could also float, as in T5R. P2's coordinate planes were typically on the eyelids (Figure 3 and Table 1). P10 Architect also had the coordinate plane on the eyelids in T1B (Table 1). In T6D, the cone was placed “with its tip touching my nose.” Similarly, for P2, in T6S, the cone rotated in pitch about the vertex, which was on the bridge of the nose (Figure 8).

Although P10 Architect's imagery floated in 22 cases (at paper distance in 18 cases and on eyelids in 4), it was rooted to architecture—ceiling, wall, or floor—in 13 cases, including D for all tasks (Table 1). P3 Active's coordinate planes appeared on a wall or blackboard six times, in S and R. P7 Vivid's cylinders in T5 were cans on the kitchen counter (Figure 6), and P9 Feeling's cones in T6 rested on horizontal surfaces. Five participants (1, 2, 4, 6, and 8) never mentioned architectural references, only body and gravity references.

P9 lacked P10's and P7's clear Euclidean relationships among architecture, the body, and gravity. Because P9 Feeling's imagery was somatosensory, the body both sensed and served as spatial reference. In T0B, P9 explained: “I don't see pictures in my head. It's more a kinaesthetic sense, like something I can reach out and feel.” However, as discussed in 3.1, P9 had a strong sense of the interactive creation of imagery. For example, when asked for more details about the parabola in T1B, P9 said: “It's there to be used, but you haven't asked me to engage with it yet.” In T0R, the plane was parallel to the ground near the left shoulder, like standing by a chalkboard “so I could engage without getting in someone else's way.” When reaching for a coordinate plane with the mind's arm, the plane tended to be a segment of a sphere (Figure 3). In contrast, in T3S, P9 traced the axes and graph of the hyperbola with virtual or tiny head movements. P9 considered the plane to have no position, perhaps meaning no Cartesian position. It would make sense that it was in a projective space, with projective but not Euclidean position. In T6B, the cone was “resting on a solid, horizontal surface.” When asked where it was, P9 responded: “Figuring out perspective wasn't something it was designed to do. When I tried, I felt like Picasso really quick.” Nonetheless, P9 could “feel” that the image was a cone and, further, that the shape changed, becoming swept, with curved-in sides, as the spiral wound upwards. How the mind's hands were attached to the body is not clear. For three other participants, the perspective in T6B was not clear: P4, P7, P10 (Figure 9). Three (P1, P6, P11) had clear, external perspectives. Two (P3, P5) solved the perspective problem of T6B by setting the cone above the eye; however, P5 could not get the spiral to get smaller as it wound up the cone. P2 had difficulty imagining the spiral, which took a “somewhat distorted path.”

As P9 wrapped the spiral around the cone in T5R, it became “more definite.” Before that, even though mathematically it was a cone: “I don't know whether it's skinny and tall or short and squat,” and “I don't know whether it makes spatial sense.” The active manipulation of wrapping the spiral cooperated with the settling of the proportions of the cone. Manipulation also made a difference for P4 Multiple, who visualized the generic coordinate plane as a “bunch” representing an equivalence class. While the coordinate planes typically faced the body, P4 repeatedly said their position was intentional, for example in T0L: “I'm willing them to move with me. They could be anyplace.” In T3, participants were asked to follow the graph from negative to positive x. P4 started in B by graphing point by point. “I'm graphing on the main one. The other ones follow.” In the next position, R: “Now it's constructed, so it can't be unconstructed. I'm just following it with my eyes.” In R, L, D, and S, the graphs turned with the body, and the mind's eyes followed the graph on the “main one” in front. “If I can't see it, I can't manipulate it, right?” P4 was asked to repeat position D, leaving the graphs above, that is, behind the body. “I can take the generic one in front of my face and manipulate it. Then the ones in back change. Or! (Laughter) Face down, I imagine my body turned over, so I can manipulate the graph in front of me.”

As discussed in 3.2.1, coordinate planes were typically in front of the face or body and body-parallel, but sometimes roll-rotated in R or L or pitch-rotated in D. In a sense, the plane is attached to the body or, equivalently, made available for manipulation, as expressed by P4.

In four cases for P3 Active, the coordinate plane was attached both to the eyes and to a wall or horizontal surface (Figure 10). In T2S, “On wall again, as before. However, as my eyes move (closed), under lid, plane moves with them, down to table top.” In T3 and T4, similar cooperative movements of the plane occurred with eye movements, from one architectural grounding to another. Similarly, P11 Precise's cylinder in T5B moved with hand movement.

The experimental tasks—imagining, following a graph (T3,4), wrapping a spiral (T5,6)—resemble many mathematicians' everyday work. They image and manipulate imagery (Sfard, 1994). Students learn to check that a graph is of a function and to be aware of the slope of the graph with respect to the axes (Vinner and Dreyfus, 1989; Tall, 1991,7). Such skills involve actively engaging with a graph, not merely picturing it.

#### 3.2.3. Mental Body Movement Mostly Respects the Geometry of Normal Movement

Movement of the mind's eye, of attention, of the mind's arm, and even movements of the “self” in engaging with imagery conform mostly to the geometry of normal vision and arm movements. The world of imagery is in many ways a simulacrum of the sensorimotor world (Farah, 1988; Kosslyn et al., 1995, 1999, 2004; Binkofski et al., 2000; O'Craven and Kanwisher, 2000; Tranel et al., 2003; Slotnick et al., 2005; Lotze and Halsband, 2006). In other words and from the opposite perspective, mathematicians use sensorimotor neural mechanisms to manipulate representations of the issues they are considering.

We have seen in the previous sections that the imagery is typically in front of the body, where the eyes and hands operate best. Further evidence for visual geometry comes from examples of what participants could “see.” Even though their eyes were closed, the ability to “see” the internal imagery with the mind's eye typically followed the geometry of eyes-open vision. P11 Precise uses visualization habitually in mathematical work, placing imagery at an angle to make it more “visible”; P11 did the same in wrapping cylinders and cones with spirals. Other participants described spirals as “invisible” or “disappearing” because they were behind a cylinder or cone (P7 T6D; P1 T5D; P2 T6B). P2 Rotator made all cylinders and some cones (R,L) transparent to “see” to wrap the spiral as it passed behind. In T6S, the cone was placed with its tip at the bridge of the nose and the axis at various angles from there, evidently so that the spiral could be “seen” (Figure 8). P3 Active made the cone in T6B transparent to see the spiral, then it was placed exactly overhead to see the whole spiral, as for P5 In-plane (Figure 9).

Another option, when the spiral went behind, was to view it with “awareness.” P1 Fluid, who followed this strategy throughout T5-6, remarked about T6L “I followed the spiral with my sight in front and awareness in back.” Awareness or attention, in this spatial sense, is closely related to eye movements (Corbetta et al., 1998; Buschman and Miller, 2007; Zhao et al., 2012; Johansson, 2013).

A variation occurred when the cylinder was especially close in T5B: P1 Fluid said, “The cylinder itself didn't move, but 'I' moved to the left as the spiral did, as though I needed to be able to see better when the spiral went around the corner because I was so close to the cylinder.” Similarly, P7 changed perspective in T0R (3.2.1).

In Tasks 3,4, participants were asked to follow the graph from negative to positive values of x. In most cases the graph was in front of the face, as was the coordinate plane in Task 0 (3.2.1; Figure 4 and Tables 1, 2). As described in 3.2.2, P4 always found a way to face the imaginary body toward the imagery, as would be most convenient for normal vision and hand movements.

Instead of the mind's eye, P9 Feeling used the mind's hand and arm. In T0L, the coordinate plane was parallel to the ground at couch level, “so I can write on it with my right hand.” Face down in T0D, the “plane wanted to be above my head” but compromised at 45°, by the forehead (Figure 4). P9 got up quickly, saying, “I felt uncomfortable, like I couldn't get to it very well.” P9 used the mind's arm in T1,2 and T4L. In most of T3-6, P9 traced graphs or spirals with head, eye, or attention movements, emphasizing that it was never a matter of seeing with the mind's eye. In T5B,R, the spiral kept spinning at the top, when the cylinder was wrapped. Next, in T5L, P9 imagined following the spiral with the mind's arm, and there was no spinning afterward. The cylinder in T5D was “quite a ways away; it's hard to experience distance.” Perhaps eye movements as a somatosensory grasping of the spiral felt like they could cover the distance to the cylinder and, in T6D, the cone. However, the geometry of eye movements may not provide distance, as virtual hand movements do, but only a projective geometry.

Both wrapping the spiral and following or graphing a curve involve engagement with imagery, as a mathematician would do when manipulating imagery in thinking. In most cases, the imagery was in front of the body where it could be viewed and often where the hands can reach (Figure 8). This area of overlap between eye and hand movements, the “manipulation zone,” is of particular importance for human engagement with tools and other objects (Graziano et al., 2002). Even when it was farther, as in P9T5D and P11T2-4D, it was in what Previc (1998) calls the “focal extrapersonal.”

#### 3.2.4. Imagery Can Accommodate the Body

Not only do the mind's eye, attention, and mind's arm move in peripersonal space like the real eye and arm, but also the imagery accommodates sensorimotor geometry, against or without the conscious intention of the imager. The appearance of imagery parallel to the body (Figure 4 and Table 2) allowed the usual relationship between graphs and the mind's eye.

Instead, P5 In-Plane started out with the body being the coordinate frame, so that eye movements were in the coordinate plane (3.2.2; Figure 7). In T1B, “I move my eyeballs and feel the need to kinaesthetically attempt to place myself, though this is disorienting.” In T2B, the imagery seems to place itself autonomously to accommodate P5: “It feels like someone drew it on a piece of paper and put it in front of my eyes...” Viewing the graph is now “easy” with it outside the body, in reach of the mind's eye that moves like the physical eye.

The direction of gravity changes sensorimotor convenience, even for the mind's eye and arm. In R,L, P6 Learner always saw the coordinates, cylinder, or cone at a diagonal between body upright and gravitational upright. Such a diagonal was not uncommon (Figures 1, 3, 8 and Table 1) and may place imagery for more comfortable movements of the mind's eye (3.2.1).

P1 Fluid's imagery slid, which avoided the need for the mind's eye to move along the axes (Figure 5C). In T0R: “The axes weren't 'in a place', i.e., the position of the origin relative to straight-forward depended completely on what I was focusing on, and I couldn't really picture the axes without focusing on a particular aspect of the axes. If I focused on the origin, it was at center, but the position was very fluid, as though the axes didn't have a position but I was just observing them more abstractly in 3D.” The *y*-axis was face-parallel, so the primary direction of mind's-eye movements would have been gravitationally vertical, as discussed in 3.2.1. By sliding, P1's imagery solved the problem “where is up?” in R and L without discomfort to the mind's eye.

In T1,2, P1's graphs centered themselves. In T1, the axes shifted down when the graph was added and zoomed larger and smaller, and in T2, the axes moved left and down, except for T2B, the last case. Such motions would center the graph more in front of the mind's eyes. However, in T1L (and later in T4L), there was an additional motion: the “*x*-axis didn't stay in my frontal plane but rather tipped back and forth relative to gravity, i.e., toward and away from my right and left sides” (Figures 3, 5D). A possible explanation is that the plane was tipping to give better views along the *x*-axis (Figure 5D). With the graph, the axes shifted down, as in T1B, presumably to center the image to the mind's eye. In T1L, the axes gradually stabilized over 3 min. “I felt as though my brain wanted to move up on the graph where the graph was wider so that my brain could grab onto the side of the graph in order to stabilize it and keep it from wobbling.” It is not clear whether the wobble is helpful to the mind's eye; there is a disagreement between imagery and imager. In T4R, the position of the coordinate plane was even more fluid: “Axes by themselves were straight and orthogonal but their orientation and position seemed quite fluid. They stayed within 45 degrees of my frontal plane, and the *y*-axis didn't tilt past maybe 20 degrees of my head-vertical, but otherwise they just seemed like an object in 3-D where position and orientation are immaterial.” P1 Fluid's graph stayed in its correct position in the axes, undistorted, and the coordinate frame moved relative to the body.

The cooperative movement of P3 Active's coordinate plane with eye movements in T2S, T3R, T4R, and T4L kept the plane in the gaze direction (Figure 10). The whole plane moved, rather than keeping areas of interest straight ahead.

P9 Feeling's coordinate plane in T1L came into being as a segment of a sphere, in reach of the mind's arm (Figure 3). It was explicitly a segment of a sphere in T1R and T2L. Other planes may also have been, without P9 mentioning it; the geometry was often difficult, as discussed in 3.2.2.

Imagery often placed itself conveniently for the mind's eye in T5,6 (Figure 8). For example, in T5S, P2 Rotator found: “The cylinder is oriented vertically, parallel to the spine, inside my head. I can 'see' both top and bottom.” In T5R, the cylinder is outside the head but “curved into a C-shape, so that both top and bottom are visible.” All of P2's cylinders were transparent, so that the spiral could be seen through the cylinder. Although P2's choice of placement near and inside the head, as if the mind's eye is inside the head, was unusual in this sample, placement for visual convenience was common (Figure 8 and Tables 1, 2).

As the mind's eye and mind's arm move in peripersonal imagery space, mostly respecting normal sensorimotor geometry, the imagery forms and places itself reciprocally in peripersonal imagery space for the convenience of the mind's eye and arm. That convenience changes with body position and gravitational direction, and both imager and imagery adapt. In most cases, the imaging process separates the imager from the imagery, with the mediation of the mind's eye and arm.

This section has shown evidence for a sensorimotor foundation to the mathematical imagery. Such a foundation relates mathematical imagery to the assigned imagery in previous sensorimotor studies (Mast et al., 2014). To summarize the evidence: the sensorimotor foundation of the mathematical imagination is displayed in coordinate plane orientation (3.2.1), relationship to the body (3.2.2), movements of the mind's eye, arm, and awareness like those of the physical eye and arm (3.2.3), and accommodations the imagery makes to the body (3.2.4). The variation in imagery orientation with body position is clear in Task 0, where the coordinate plane is typically in front of the face, but may roll in R and L, usually toward gravitational vertical (3.2.1, Figure 4 and Tables 1, 2). Imagery is almost always tightly related to the imager's body, whether to see, with the body as coordinate system, or to manipulate (3.2.2, Tables 1, 2). P9 Feeling provides an interesting exception of a fractured space in T6B, where both P9 and the cone were resting on horizontal surfaces, P9 could access the cone, but the geometrical relationship between the body and the cone was “not something [the imagery] was designed to do” (3.2.2, Figure 9). Awareness sometimes can see the far side of a solid mental cylinder or cone (3.2.3). An exception to placing the coordinate plane in front of the face so that the mind's eye and arm can reach it is in D, apparently to avoid placing the coordinate plane within furniture (3.2.1). The imagery itself cooperates autonomously to place itself for convenient access, showing itself to be in sensorimotor relationship (3.2.4, Tables 1, 2).

### 3.3. Relationship of Abstract Mathematical Imagery to the Concrete

Using the sensorimotor system separates the imagery from the imager, giving the imagery—even though it is part of the imager—properties of an object. It is a short step to giving the imagery concreteness. The mathematicians both produced and resisted concreteness (3.1). When asked in T5D what the spiral was made of, P9 said, “I don't know. It feels like asking What color is square? or What color is 2?” Concreteness was seen as an intrusion, often amusing. For example, in T5S, P1 Fluid said, “The spiral/cylinder situation was the same as in [D] except that the spiral went around about three times and was greenish (“yellowish-green”?) and fuzzier. (I'm laughing now.)” Besides describing mathematical objects as concrete objects, participants treated them as if concrete, described them acting as if concrete, and gave imagery concrete substrates (Table 3).

**Table 3:** Concreteness and autonomy.

**Participant**	**Color, Texture**	**Concrete**	**Movement of imagery**	**Autonomy**
P1 Fluid	14 mostly T4-6; 15 black, mostly T1-3; 5 textured	grab T1L; in T5,6: 6 is object; 4 drawing	convenience for mind's eye; T5R drifting away; T6B moving substrate; T6L widening cone	Pervasive: movement of imagery; colors; materials; textures; placement
P2 Rotator	1 T6B; 6 transparent in T5-6	0	T0S, T1B,S,T2L drifting; T1R,L,T2B,R,T3,4L graph distorts; T6B path distorts; T6R cone moves	Pervasive: placement, drifting, distortion
P3 Active	7 T5-6	10 arch ref; is object T6D	plane moves with eyes; T4-6 relating to function	Pervasive: placement, movement, distortion of graphs and axes;
P4 Multiple	1 transparent T6S	15 willing; is object T6; substrate T6	others follow	Almost none
P5 In-plane nv	0	4 substrate; falling T1L;1 simile; 3 arch ref	T1L graph falls down; T5L rotating same direction	Few but dramatic: movement; tension; placement
P6 Learner	0	2 arch ref	0	Placement; tension
P7 Vivid	0	6 substrate; 8 arch ref; 9 is object in T5,6	relating to function	Pervasive: movement; placement; objects
P8 Straight-ahead nv	0	0	T2 graph swoops around	Almost none
P9 Feeling nv	1? T1B	3 substrate; 3 simile; 3 couch conflict	T4S graph wiggling; T5B kept spinning at top; T6B string slow, then fast; cone swept	Minor but vivid: movement; placement
fP10 Architect	6 T3D; others in T5,6	19 arch ref; 15 substrate; 4 simile	T4R graph tilts as head follows graph; T6S cone rotated	Medium: placement; movement
P11 Precise	6 T2-4D, T6; 7 black/grey/white T0,1D, T2-4S, T3,4B	4 (+4?) couch conflict; 7 simile; 2 is object	T1R parabola arm asymptoting to gravity; T5B moved with hand	Minor: movement; objects

*Under “Color, Texture” numbers count instances of color. Under “Concrete,” “is object” describes imagery being a concrete object, whereas “simile” describes imagery being like a particular concrete object. arch - architectural Under “Movement of Imagery” are movements for sensorimotor convenience, with respect to gravity, with mathematical meaning, and that remain unexplained. “Autonomy” lists actions imagery has taken without or against the intention of the imager*.

In many cases, participants looked to see where the imagery was and what it was doing, tacitly giving it autonomy (Table 3, Autonomy). Since they construed the imagery as a separate entity, it made sense to interact with it.

Every mathematician at some point gave imagery concrete properties or autonomy, even P8 Straight-ahead, whose parabola in T2 “swoops around.” P4 Multiple, who resisted even choosing a particular placement (3.1), visualized ice-cream cones in a holder in T6. P6 Learner's imagery placed itself autonomously; in R,L, it placed itself on a diagonal that sometimes caused tension (3.2.1). Overall, the concrete aspects and autonomy gave the mathematicians' imagery exuberance. For example, P11 Precise's graphs in T2-4D were deep in the earth and rainbow-colored on a dark background.

Five participants' imagery had color (Table 3), and P1 Fluid's had texture. In T5,6, participants said that imagery was an object, such as a pipe or a traffic cone (Table 3). P9-11 said that imagery was like an object (Table 3). For example, P11's spirals were two-dimensional and normal to the cylinder or cone, “like a scarf.” Six participants (P1,3,4,7,10,11) had more concrete imagery in T5,6, likely because we manipulate concrete objects, although also because cylinders and cones invite concrete imagery. In some cases, the manipulation effects depended on position. For example, P6 Learner commented in T4,5R.L that focus was required, whereas the other positions were easy, and in T6R,L that the cone was “more strongly inclined” toward gravitational vertical. It was only in R,L that P1 Fluid's cylinder and cone moved: the cylinder moving down and right in T5R, and the cone flattening out in T6L.

Even abstract mathematical imagery was treated as if it were concrete. In T0D, 3 participants' (P4,7,9) coordinate planes were rotated over their heads or behind, so that they did not occupy the same space as the supporting furniture, even though that placement put them out of normal visual range. With eyes closed, mental imagery occupies peripersonal space. At the same time, peripersonal space is filled with concrete objects, such as furniture, one knows are there but can't see or feel. Eight participants immersed mental imagery within the mental awareness of furniture in T0; three (P4,7,9) avoided overlap. In the other tasks, the three avoided conflict in some way: by placement, by tension, or (P9) by changing from virtual arm movements to movements of attention or of the eyes (3.2.3). Requests to follow a graph (T3,4) or wrap a spiral (T5,6) caused P4 a conflict in D between avoiding imagery/furniture conflict and the need to have at least one example of the imagery in view, either in front or to the side, for manipulation (3.2.2).

P11 expressed the same conflict in a different way. In T1R, as in T0R, the axes were up to the left, so they wouldn't hit the couch, and rotated 45° so that the *y*-axis was between body- and gravity-parallel. In addition, the graph came out “skewed as if asymptoting to the *y* axis,” that is, asymptoting to gravitational vertical, like a plant growing upward. The plane itself is skewed toward the gravitational vertical, and the graph is separately and further affected by the gravity. Gravity affecting imagery shows imagery behaving like a concrete object, with mass, or autonomously, reacting to gravity. P2 also had an arm of a parabola reach up in gravity, P5's parabola fell down (3.2.2; Figure 7), and P1 speculated in T4R that the plane was pulled by gravity. Unlike P4 Multiple's other imagery, the image of the cone was single at first, because “a cone can't stand.”

Another interaction between the concrete surround and imagery was as a substrate (Table 3). The most dramatic was P10 Architect's use of the corner where the wall meets the ceiling as a *y*-axis in T0L (Figure 4). The *x*-axis was also on the ceiling. In 14 out of 35 task/positions, P10's imagery used an architectural substrate: ceiling, floor, or wall (Tables 1, 2). In T5B, P10 said: “A large cylinder comes up from the ground, like a column rising to the ceiling.” Each D image was on the floor. P7 Vivid's cylinder in T5B is a can (imagery) on the real, visualized kitchen counter in the next room, “in a position where it won't roll.” Wrapping the spiral became play with concepts of infinitesimal and infinite (3.5). The can became infinitely tall. In T5R, the can “goes through the floor, down through my neighbors' kitchens, and so on.” P4 Multiple's cones in T6 were in an ice-cream cone holder, so that they could be an equivalence class and not fall over. P9 Feeling's cone in T6S stood on a square base, and in B on a horizontal surface. P1 Fluid's wooden cone in T6B stands on a horizontal base that is “wavy, moving, and bluish” (Figure 9). In S, the cone had “its base on a flat surface (so sort of 2D as a drawing, but still known to be 3D).” P10 Architect's cylinder in T5D was at first “2-dimensional, as if drawn on ... poster board” on the floor. “However, as I force myself to think three dimensionally, the cylinder rises from the ground in front of me.” P3 Active's cone in T6S “won't stay the same size!” After a struggle, P3 draws it on a blackboard. P7 Vivid's cone in T6S is drawn on paper, but the spiral wrapped in three dimensions, coming through the paper.

Six participants had physical-like conflicts with imagery, in which autonomy and concreteness shaded into each other. P1 Fluid's cylinder in T5R kept slipping aside and being brought back mentally (Figure 8). The tan wood cone in T6L “was trying to get wider but I had to stop it so that it wouldn't just flatten out.” This widening out recognizes that mathematically there are a range of cone shapes, like P4 Multiple's equivalence class (3.1). P1 wanted to “grab ... the graph” in T1L (3.2.4). P3 Active had lively struggles with imagery. For example, in T5S, the cylinder became a cone; P3 forced it open. In T6D, P3's first imagery in T6D was “sorta red.” The abstract imagery was lost, and it was difficult to wrap a spiral. “The only thing that works is turning it into a silver earth-boring drill, with a pre-cast spiral already in place.” P5 In-Plane also had lively active and passive interactions with imagery and the imagery space. In T1R, “I feel like I am facing the 'wrong way.' I feel like I am upside down or like I am using my non-dominant hand to write...” P5's struggled in T6B,S to tighten the spiral as it rose to the cone tip. As P9 Feeling's string spiral wrapped the cone in B, it sped up, and the cone narrowed, so that the sides curved into a swept shape. P9 could keep the sides straight “with attention, but it's really slow.” P7 Vivid, in T5R, found the spiral widening, “and I ... keep having to push it in, ... like a spring.” In T6, starting from the point, P7 found the wire spiral taking “longer and longer” as the cone widened. In B,R, it became “harder” and even “arduous.” In L,D it became “slower,” “the cone's missing,” so that the “ropes” of the spiral, in their “spinning motion” became “like the clouds in the tornado.” The “tension” that P6 learner feels in R,L of most tasks is similar, without concrete simile (3.2.1). In contrast, P4 places passive imagery by “willing” (3.2.2; Table 3).

### 3.4. Coordination of Imagery Components

Working with imagery, a mathematician may move one component of a mathematical structure relative to another or to a coordinate plane. An architect or remodeler may perform a similar mental operation, imagining where best to place a door or stairway, by mentally moving it relative to the house. Arguably, the composition and inspection tasks in Mast et al. (2003) may assign a similar mental action. Other times, visual imagery works with semi-autonomy, proposing solutions. This section examines the coordination of separate components within imagery.

Participants displayed separation of components to varying degrees. Even in T1, where no following or wrapping of imagery was requested, P7 Vivid's body position, coordinate plane, and parabola moved independently (Figure 11). In contrast, P6 Learner only distinguished self and imagery.

P2 Rotator's coordinate planes were typically in the plane of the eyes, on the surface of the body (Table 1). However, in T1L, the body and plane separated: the plane slipped around the head to the left, downward in gravity toward horizontal (Figure 3). The parabola moved in the plane, with one arm elongated toward gravitational vertical. So P2 separated self, plane, and parabola, with separate effects on the plane and an arm of the parabola.

P5 In-Plane identified the plane with the body (3.2.2) in early tasks. In T1B,L,D, the plane is identified with the body, and the parabola is separable from the plane (Figure 7). In T2B, the parabola and plane form a unit, separate from the body. P5 has changed from body/plane vs. graph (in T1B,L,D) to body vs. plane/graph (in T2B) (slash indicating a unit).

Tasks 3 ,4 ask participants to follow the graph negative to positive—a request to separate elements. In T3, P4 Multiple realized that the graph had to be in front of the body to follow or “manipulate” it (3.2.2). The manipulation became more challenging when participants were requested to wrap a spiral around a cylinder (T5) or cone (T6). In T5R, P4 commented: “They're hard to wrap unless they're in front and parallel to my body. ... If I leave them [gravity-parallel rather than body-parallel], it's as if I have to think around a corner.” Only in T6 did P4's imagery become susceptible to gravity (3.2.2).

Somewhat like P7's parabola gliding into place (Figure 11), P10 Architect's spiral moves into place in T5B: “A large cylinder comes up from the ground, like a column rising to the ceiling. The spiral wraps around it like a wire. My head moves slightly to follow the spiraling wire.” For five participants (P1,2,6,10,11), the spiral wrapped autonomously. P4 Multiple wrapped the spirals, P8 denoted T5,6, and the rest (P3,5,7,9) mixed spontaneous wrapping with active participant wrapping. Although five participants (P1,4,6,10,11) maintained spiral, cylinder, and cone shapes as the spiral wrapped, others experienced variations.

P5 In-plane, who doesn't habitually use internal imagery, strikes most of the themes in the relationship between cylinder or cone and spiral components. P5 found in T5B that the cylinder and spiral “don't mix ... They didn't feel related to each other.” Next, in R: “I can wrap the spiral around it easily this time. It starts at the bottom and wraps up. ... the cylinder then continued on for much longer than the original assumed length making it feel infinitely long.” In R, the wrapping starts as P5's action, but then becomes autonomous and continues, perhaps to infinity. Then in L: “The cylinder is rotating away from me. ... I cannot make the spiral go up the cylinder since they go in the same direction at the same time.” D is easy, and S is difficult.

P9 Feeling's spiral also kept spinning at the top in T5B,R. In S, the wrapping was “loose.”

In T6B,S, P5's difficulty was in making the spiral “tighten up to match the shape.” R, L, and D were easy.

P3 Active also had difficulties wrapping spirals in T6 (3.3). In L: “Cone evaporates midway,” spiral “sproings off, gets twice as large as base before it reaches the height where it would have been at the top of the cone.” In S: “Cone won't stay the same size! ... It turns into a cylinder.”

P2 Rotator's spirals tended to distort in T6. Cones were near the nose and sometimes inside the head, like the cylinders in T5 (Figures 6, 8) In B: “Difficult to imagine spiral, somewhat distorted path around cone...” In R: “Transparent cone ... As spiral is formed, cone is narrowed; as if spiral is constricting the cone.” In L: “Transparent cone ... Spiral winds unevenly around cone, but no distortion of cone.”

The narrowing of the cone in P2T6R is arguably an expression of the relationship between cone and spiral, rather than a distortion of that relationship. In T6B, P9's “string [spiral] went slow, then really fast,” expressing the change in distance and the cone “swept,” curving in at the top.

As a continuation of P7 Vivid's infinity theme in T5 (3.3, Figures 8, 9), in T6 the icecream cone becomes huge, and the lengths of the distances around the cones become larger and larger. In B: “As I go farther and farther up, it takes me longer and longer to go around. ... So it's getting more and more arduous ... the spiral is the cone ... it's like a shell ... it just doesn't ever stop.” Then in R: “It's like the icecream cone isn't different from where it was a moment ago; I've just turned. ... like a robot icecream cone ... wrapped in wire ... The cone keeps getting larger and larger. And ... the spiral sort of lags behind ... It's sort of like the cone is waiting for the spiral to finish it.”

P3 Active's spirals in T5 expressed both difficulty and relationship to the cylinder. In R: “Spiral hits closed loop attractor, and just spirals asymptotically to it. Poor spiral, never making it to the top of the cylinder.” In L: “...doesn't spiral in a geodesic. As it goes up, it drops down quite a bit before going back up.” In D: “Spiral starts at the top of my head, hits an attractor around my forehead or eyes, asymptotically spirals out. Try starting at feet. Shoots up in a few spirals, again, non-geodesics with significant drop-downs on the back-side of the spiral necessitating steep upsurges on the front side.” In S, it is drawn on a blackboard: “Spirals up nicely, except that the cylinder has become a cone! As such, spirals tighter and tighter at the vertex. I force it open, and all is forgiven as the spiral concludes its upward journey.”

### 3.5. Mathematical Ideas

The attractors and non-geodesics of P3's spirals actively expressed a mathematical idea, rather than offering sensorimotor convenience. So did P7 Vivid's infinity theme, P9 Feeling's sweeping of the cone shape as the spiral is added (3.4), and P3's cylinder turning into a cone. All participants described imagery that expressed mathematical ideas not directly requested, some more exuberantly than others.

Fixed expressions of mathematical ideas included P4 Multiple's equivalence classes of visualizations (3.1) and P11 Precise's preference to work in three dimensions (3.2.1). P6 Learner expressed the same preference more mildly. The three-dimensionality of the peripersonal space in which the imagery was created was expressed in many ways, including P5's parabola falling down (T1-2, Figure 7, 3.2.2) and P1's coordinate plane wobbling in three dimensions (Figures 3, 5, 3.2.4).

One is tempted to think of three-dimensional imagery space as a Euclidean space, as in classical physics. However, neurally it is unlikely to be so, since sensation depends on disparate senses and on processes at multiple levels of neural organization. For example, P10 Architect in T5D first imaged the cylinder as a two-dimensional drawing on the floor. “However, as I force myself to think three-dimensionally, the cylinder rises from the ground before me, so that I see its circular face approaching.” P10 has two simultaneous perspectives: “I am imagining the spiral as if I were looking at it from an angle, even though my original picture is of the cylinder directly beneath me.”

Similarly, using somatosensory imagery, P9 Feeling had two perspectives in T5B (3.2.2). Then in T5R, P9's cone lacked a fixed geometry: “I don't know whether it's skinny and tall or short and squat” and “I don't know whether it makes spatial sense.” Mathematically, it was still a cone, just not specified spatially. It became more definite as the spiral was wrapped about it. Similarly, P1 Fluid's cone widened (3.3, Figure 8 T6L).

Just as a cone and a circle have a specific shape however tall or squat the cone or however large or small the circle, a parabola has a precise shape, no matter how broad or skinny it is (using P9 Feeling's descriptors). P7 Vivid's parabola could glide into place on the coordinate grid (3.4; Figure 11) because it did not need coordinates to specify the shape. For example, in T1D, P7 said: “There's no calculation, there's no square involved, it's just along that shape.” Mathematically, P7 is expressing the fact that the parabola has a geometric shape independent of its expression as a quadratic equation. Also sensitive to the shape, P1 and P2 remarked that their parabolae were not shaped correctly. For example, in T2R, P1 said: “The shape wasn't quite correct, as the vertex was too pointed and I couldn't get it to be the right curviness.”

**Figure 12:**
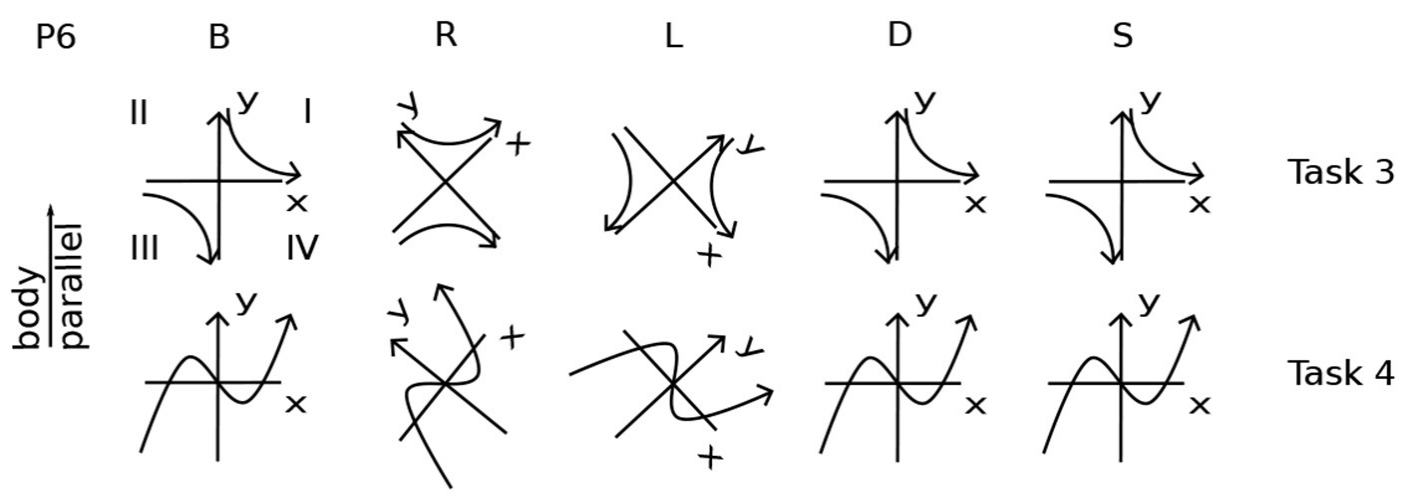
Computer-graphic approximations of p6 Learner's Sketches in T3,4. Quadrants are labeled conventionally with Roman numerals I-IV. Another convention participants used is to label them the 1st quadrant through the 4th quadrant. P6's sketching convention is that body vertical is vertical on the page.

The graph of *y* = 1/*x* in Task 3 consists of an hyperbola in the third and first quadrants (Figure 12). Like the parabola, the hyperbola is a familiar shape, so it can just appear. For example, P7 Vivid said in T3B: “I'm not thinking of drawing it...; it's just there.” The axes and curves came into place autonomously, with the two halves of the curve separate. After drawing the hyperbolae in B, P5 In-plane pictured the graph all at once in R: “I can only focus on one part at a time but I know where they are in orientation to each other as I switch between the two pictures.” However, in L, the two parts “are floating and not staying in place correctly.” Similarly, in S and B, P2 Rotator lost sight of the left half of the graph in moving right. In contrast, for three participants (P3, P4, and P6), the two parts were together, but the graph blinked or the attention jumped at asymptotes at the *y*-axis. P6 Learner said in T3B: “hard to traverse curve L to R across *y*-axis in my mind,” and in D and S, “hesitates at jump.” The “jump” in T3 is an expression of both the mathematical asymptotes and the visual difficulty of jumping from one to the other.

For P2, the graph in T3 became distorted when added to the axes. Like P7 Vivid (3.4; Figure 11), P3 Active had a clear and attentive awareness of the separateness of coordinate plane, graph, and their position in space. However, the shapes became distorted. In T3B: “moving further positive along the *x*-axis causes the *x*-axis to sag and droop.” In R, the graph “coming down from infinity for positive x, just decides to head straight down and not move away.” In L, “*x*-axis sags and bends, but 1/x crosses sagging *x*-axis, and becomes concave down, and just drops.” In D, “graph goes concave up instead of asymptotic to *x*-axis in first quadrant.” In S, the hyperbola in the third quadrant, when it should be asymptoting toward negative infinity, “bends away from *y*-axis.” It is not clear what the causality is in these visualizations, but P3 is sensitive to the asymptotes and the correct shape of the hyperbola.

The function in Task 4, x(x2-1), is easy to factor into x(x-1)(x+1), giving the zero-crossings –1, 0, and 1 (Figure 12). Two mathematicians explicitly factored (P10, P11). Five were confident from the beginning of the overall shape and zero-crossings, so evidently factored (P1, P2, P3, P6, and P8). The standard cubic shape can be morphed as a whole to fit the zero-crossings. P9 Feeling took a more experiential approach, starting from the left, pointing with the eyes, but “At first I wasn't sure where it would be. ... Consciousness expanded as I went,” so that the whole graph became clear. P7 Vivid's imagery was the most detailed, giving close attention to the shape of graph segments. P7's experience was of the graph as it curved, dipped, and rose, rather than of a picture of the whole graph on axes.

P3 Active responded to the up-and-down shape of the cubic, saying in T4B: “the whole graph emerges, then starts dancing up and down, oscillating fairly wildly.” Although P3 Active is the clearest about oscillatory movement of the cubic graph, three other participants mentioned something similar: P7 Vivid mentioned “choppy” and said axes became irrelevant, P9 Feeling said the graph wiggled and came separate from the axes, and P10 Architect in T4B said “Here I finally feel movement! I do a little bump/bounce up/down with my head as I follow the graph from left to right.”

## 4. Discussion

The sensorimotor foundation of the mathematical imagination is not obscure, but easily accessible, as demonstrated in this experiment. First, in every body position, the imagery is placed and oriented with respect to the body, with variations depending on body position (3.2.1). In particular, the imagery in R,L tends to be tilted in the roll direction, whereas imagery in D tends to shift in pitch. Second, the imagery has particular relationships to the body (3.2.2). For example, the body can be the coordinate system (P5) or the imagery can be on the eyelids (P2 and P10). Third, the mind's eye and arm, along with the awareness, usually has the same geometry as the real eye and arm (3.2.3). Imagery usually has to be in front of the face to be seen and especially to be manipulated. Fourth, the imagery moves to accommodate the body (3.2.4). For example, after some struggles, P5 In-plane's imagery autonomously drew itself and placed itself in front of the eyes.

This sensorimotor nature of mathematicians' imagery relates it to non-mathematical imagery studied by manipulating the vestibular sense (Corballis et al., 1978; Marendaz et al., 1993; Gaunet and Berthoz, 2000; Mast et al., 2003, 2014). Whether visual, or somatosensory, the mathematicians' imagery primarily expressed spatial relationships rather than elaborating details of objects (Kozhevnikov et al., 2005). Autonomy has been a salient feature of mathematical imagery (Hadamard, 1945) and is detailed in the present study, I am not aware of any mention of autonomy in studies of assigned imagery (Kozhevnikov et al., 2005; Mast et al., 2014; Hegarty et al., 2018).

The variation and individuality is a major point in this study: mathematicians are not uniform, and students need not be forced to be. Mathematical correctness is sufficient uniformity. In contrast, studies intended to produce statistical differences between groups attempt to make imagery performance uniform, in order to be comparable. However, Kozhevnikov et al. (2005) show a lovely example of individuality in imagery in Study 4. Probably such studies conceal spectra of individuality such as the present study reveals.

In the present study, a cornucopia of imagery forms emerged, without being directly queried. Each participant's imagery was individual, while showing family resemblances (3.1). Throughout, the imagery placed itself and moved autonomously, with wide individual variation. There was a varying relationship to the concrete (3.3), which raises questions. What is the nature of a parabola that falls in gravity (P5, Figure 7) or that has an arm that reaches up in gravity (P2, Figure 3, 3.1; P11, 3.3)? P7's parabolae glide into place on the coordinate grid, as if obeying a different force as clear as gravity (Figure 11).

These Alice-In-Wonderland imagery phenomena are endemic to mathematics, as discussed by Núñez in his delightful essay on the metaphorical motions of numbers (Núñez, 2006). Núñez suggests that mathematicians' imagery follows unconscious habits and that sometimes those habits contradict the mathematics itself. As he points out, a road or a function doesn't go anywhere; it's static. However, counter-factual imagery may be useful to creativity.

Sriraman (2004) documents the use of imagery for mathematical creativity. There are multiple ways imagery may be used creatively in mathematics, remembering that not all mathematicians use imagery and that imagery can be used non-creatively. One way is for imagery to autonomously suggest options. Such a suggestion can become a flash of insight, from out of the blue, when the imager recognizes its significance. The autonomy of imagery is amply illustrated in this experiment. Autonomy of imagery likely arises at least partly from automaticities built into mathematicians' habits of imagination. Fluency in reading, a similarly well-practiced skill, includes an automatic correction of misspellings (Potter et al., 1993). Such automaticities facilitate fluent performance in reading, mathematics, or almost any skill. Perturbing mathematicians' imagery habits by having them turn in different orientations to gravity and different body positions reveals some of the automaticities they have built in.

Another way to use imagery for creativity is as an expressive medium, to communicate with others (Sfard, 1994; Ochs et al., 1996). Communication opens the way for creative collaboration.

Another is as a supple medium in which to express possibilities to oneself. Use of imagery as a medium would lead to such imagery skills as demonstrated by several of the participants, for example, the ability to coordinate multiple imagery components.

The present study is about the sensorimotor underpinnings of the mathematical imagination, not about the sensorimotor underpinnings of mathematical structures. For example, the concept of the integers—the numbers both positive and negative—may arise from right-left body symmetry and its related neurobiology and mental habits (Blair et al., 2012). More broadly, Lakoff and Núñez (2000) propose that mathematics arises from the metaphor of embodied ideas. Mathematicians commonly have imagery shorthand for an abstract mathematical concept, called a concept image (Tall and Vinner, 1981). It is a short step from imagery based on the sensorimotor world to concepts based on the sensorimotor world.

## Data Availability Statement

The raw data supporting the conclusions of this article will be made available by the authors, without undue reservation.

## Ethics Statement

The studies involving human participants were reviewed and approved by the Institutional Review Board, Human Subjects Research Review Committee, Portland State University. The patients/participants provided their written informed consent to participate in this study. Written informed consent was obtained from the individual(s) for the publication of any potentially identifiable images or data included in this article.

## Author Contributions

GM thought of the experiment, gathered and analyzed the data, made the drawings, and wrote the manuscript.

## Funding

The Publication Fee was supported by Gerhard Magnus, Phoebe Warren, Wendy Ellison, Elizabeth Robbins, Nancy Church, Laura Smolkin, David Summers, Sarah Morgan, Larkin Plaeger-McCollum, Hariharan G. K., and an anonymous donor.

## Conflict of Interest

The author declares that the research was conducted in the absence of any commercial or financial relationships that could be construed as a potential conflict of interest.

## Publisher's Note

All claims expressed in this article are solely those of the authors and do not necessarily represent those of their affiliated organizations, or those of the publisher, the editors and the reviewers. Any product that may be evaluated in this article, or claim that may be made by its manufacturer, is not guaranteed or endorsed by the publisher.

## References

[B1] Barsalou L. W. (2008). Grounded cognition. Ann. Rev. Psychol. 59, 617–645. 10.1146/annurev.psych.59.103006.093639 17705682

[B2] Barsalou L. W. Simmons W. K. Barbey A. K. Wilson C. D. (2003). Grounding conceptual knowledge in modality-specific systems. Trends Cogn. Sci. 7, 84–91. 10.1016/S1364-6613(02)00029-3 12584027

[B3] Bigelow R. T. Agrawal Y. (2015). Vestibular involvement in cognition: visuospatial ability, attention, executive function, and memory. J. Vestibular Res. 25, 73–89. 10.3233/VES-150544 26410672

[B4] Binkofski F. Amunts K. Stephan K. M. Posse S. Schormann T. Freund H-. J.. (2000). Broca's region subserves imagery of motion: a combined cytoarchitectonic and fMRI study. Human Brain Mapping 11, 273–285. 10.1002/1097-0193(200012)11:4<273::AID-HBM40>3.0.CO;2-0 11144756 PMC6872088

[B5] Blair K. P. Tsang J. M. Schwartz D. L. (2012). “The bundling hypothesis: how perception and culture give rise to abstract mathematical concepts,” in International Handbook of Research on Conceptual Change II, ed. S. Vosniadou (New York, NY: Taylor and Francis) Chapter 17, 322–340.

[B6] Buschman T. J. Miller E. K. (2007). Top-down versus bottom-up control of attention in the prefrontal and posterior parietal cortices. Science 315, 1860–1862. 10.1126/science.1138071 17395832

[B7] Corballis M. C. Nagourney B. A. Shetzer L. I. Stefanotos G. (1978). Mental rotation under head tilt: factors influencing the location of the subjective reference frame. Perception Psychophys. 24, 263–273. 10.3758/BF03206098 704287

[B8] Corbetta M. Akbudak E. Conturo T. E. Snyder A. Z. Ollinger J. M. Drury H. A.. (1998). A common network of functional areas for attention and eye movements. Neuron 21, 761–773. 10.1016/S0896-6273(00)80593-0 9808463

[B9] Elterman R. D. Abel L. A. Daroff R. B. Dell'Osso L. F Bornstein J. L (1980) Eye movement patterns in dyslexic children. J. Learn. Disabilities 13 16–21. 10.1177/002221948001300104 7373140

[B10] Farah M. J. (1988). Is visual imagery really visual? overlooked evidence from neuropsychology. Psychol. Rev. 95, 307–317. 10.1037/0033-295X.95.3.307 3043530

[B11] Ferrè E. R. Berlot E. Haggard P. (2015). Vestibular contributions to a right-hemisphere network for bodily awareness: combining galvanic vestibular stimulation and the ‘rubber hand illusion.’ Neuropsychologia 69, 140–147. 10.1016/j.neuropsychologia.2015.01.032 25619847

[B12] Fuson K. C. Wearne D. Hiebert J. C. Murray H. G. Human P. G. Olivier A. I.. (1997). Children's conceptual structures for multidigit numbers and methods of multidigit addition and subtraction. J. Res. Math. Educ. 28, 130–162. 10.5951/jresematheduc.28.2.0130

[B13] Gaunet F. Berthoz A. (2000). Mental rotation for spatial environment recognition. Cogn. Brain Res. 9, 91–102. 10.1016/S0926-6410(99)00038-5 10666561

[B14] Graziano M. S. A. Taylor C. S. R. Moore T. Cooke D. F. (2002). The cortical control of movement revisited. Neuron 36, 349–362. 10.1016/S0896-6273(02)01003-6 12408840

[B15] Greiffenhagen C. (2014). The materiality of mathematics: presenting mathematics at the blackboard. The Brit. J. Sociol. 65, 502–528. 10.1111/1468-4446.12037 24620862

[B16] Hadamard J. (1945). An Essay on the Psychology of Invention in the Mathematical Field (Princeton, NJ: Princeton University Press).

[B17] Hegarty M. Burte H. Boone A. P. (2018). “Individual differences in large-scale spatial abilities and strategies,” in Handbook of Behavioral and Cognitive Geography, Chapter 13, 231–246. 10.4337/9781784717544.00022

[B18] Johansson R. (2013). Tracking the Mind's Eye: Eye Movements During Mental Imagery and Memory Retrieval. Lund: Lund University.

[B19] Kosslyn S. Thompson W. Shephard J. Ganis G. Bell D. Danovitch J. (2004) Brain rCBF and performance in visual imagery tasks: common distinct processes. Eur. J. Cogn. Psychol. 16, 696–716. 10.1080/09541440340000475.

[B20] Kosslyn S. M. Pascual-Leone A. Felician O. Camposano S. Keenan J. P. Thompson W. L.. (1999). The role of area 17 in visual imagery: convergent evidence from PET and rTMS. Science 284, 167–170. 10.1126/science.284.5411.167 10102821

[B21] Kosslyn S. M. Thompson W. L. Kim I. J. Alpert N. M. (1995). Topographical representations of mental images in primary visual cortex. Nature 378, 496–498. 10.1038/378496a0 7477406

[B22] Kozhevnikov M. Kosslyn S. Shephard J. (2005). Spatial versus object visualizers: a new characterization of visual cognitive style. Memory Cogn. 33, 710–726. 10.3758/BF03195337 16248335

[B23] Lakoff G. Núñez R. E. (2000). Where Mathematics Comes From: How the Embodied Mind Brings Mathematics into Being, (New York, NY: Basic Books).

[B24] Liebowitz H. W. Post R. B. Sheehy J. B. (1986). Efference, perceived movement, and illusory displacement. Acta Psychologica 63, 23–34. 10.1016/0001-6918(86)90040-5 3591434

[B25] Lotze M. Halsband U. (2006). Motor imagery. J. Physiol. - Paris 99, 386–395. 10.1016/j.jphysparis.2006.03.012 16716573

[B26] Lowrie T. Logan T. Harris D. Hegarty M. (2018). The impact of an intervention program on students' spatial reasoning: student engagement through mathematics-enhanced learning activities. Cogn. Res. Principles Implications 3, 1–10. 10.1186/s41235-018-0147-y 30585295 PMC6305681

[B27] Marendaz C. Stivalet P. Barraclough L. Walkowiak P. (1993). Effect of gravitational cues on visual search for orientation. J. Exp. Psychol. Human Percept. Perform. 19, 1266–1277. 10.1037/0096-1523.19.6.1266 8294891

[B28] Mast F. W. Ganis G. Christie S. Kosslyn S. M. (2003). Four types of visual mental imagery processing in upright and tilted observers. Cogn. Brain Res. 17, 238–247. 10.1016/S0926-6410(03)00111-3 12880895

[B29] Mast F. W. Preuss N. Hartmann M. Grabherr L. (2014). Spatial cognition, body representation and affective processes: the role of vestibular information beyond ocular reflexes and control of posture. Front. Integrative Neurosci. 8: article 44, 1–14. 10.3389/fnint.2014.00044 24904327 PMC4035009

[B30] Núñez R. (2006). “Do real numbers really move? language, thought, and gesture: the embodied cognitive foundations of mathematics,” in Unconventional Essays on the Nature of Mathematics. ed R. Hersch (New York, NY: Springer Science+Business Media, Inc.).

[B31] Ochs E. Gonzales P. Jacoby S. (1996). “When I come down I'm in the domain state': Grammar and Graphic Representation in the Interpretive Activity of Physicists,” in Interaction and Grammar, eds. E. Ochs, E. Schlegoff, and S. Thompson (Cambridge, MA: Cambridge University Press). 10.1017/CBO9780511620874.007

[B32] O'Craven K. M. Kanwisher N. (2000). Mental imagery of faces and places activates corresponding stimulus-specific brain regions. J. Cogn. Neurosci. 12, 1013–1023. 10.1162/08989290051137549 11177421

[B33] Pansell T. Ygge J. Schworm H. D. (2003) Conjugacy of torsional eye movements in response to a head tilt paradigm. Investigat. Ophthalmol. Vis. Sci. 44, 2557–2564. 10.1167/iovs.02-0987 12766057

[B34] Péruch P. Lopez C. Redon-Zouiteni C. Escoffier G. Zeitoun A. Sanjuan M.. (2011). Vestibular information is necessary for maintaining metric properties of representational space: evidence from mental imagery. Neuropsychologia 49, 3136–3144. 10.1016/j.neuropsychologia.2011.07.026 21820000

[B35] Poincaré H. (1908). (translated by Maitland F,1914) Science and Method. (London: Thomas Nelson and Sons).

[B36] Potter M. C. Moryadas A. Abrams I. Noel A. (1993). Word perception and misperception in context. J. Exp. Psychol. Learn. Memory Cogn. 19, 3–22. 10.1037/0278-7393.19.1.3

[B37] Previc F. H. (1998). The neuropsychology of 3-D space. Psychol. Bull. 124, 123–164. 10.1037/0033-2909.124.2.123 9747184

[B38] Rayner K. (1977). Visual attention in reading: eye movements reflect cognitive processes. Memory Cogn. 5, 443–448. 10.3758/BF03197383 24203011

[B39] Schworm H. D. Ygge J. Pansell T. Lennerstrand G. (2002). Assessment of ocular counterroll during head tilt using binocular video oculography. Investigat. Ophthalmol. Vis. Sci. 43, 662–667. 11867581

[B40] Seitz J. A. (2000). The bodily basis of thought. New Ideas Psychol. 18, 23–40. 10.1016/S0732-118X(99)00035-5

[B41] Sfard A. (1994). Reification as the birth of metaphor. For the Learning of Mathematics 14, 44–55.

[B42] Slotnick S. D. Thompson W. L. Kosslyn S. M. (2005). Visual mental imagery induces retinotopically organized activation of early visual areas. Cerebral Cortex 15, 1570–1583. 10.1093/cercor/bhi035 15689519

[B43] Sriraman B. (2004). The characteristics of mathematical creativity. Mathematics Educator 14, 19–34.

[B44] Sun F. Morita M. Stark L. W. (1985). Comparative patterns of reading eye movement in chinese and english. Percept. Psychophys. 37, 502–506. 10.3758/BF03204913 4059005

[B45] Tall D. (1991). Intuition and Rigour: The Role of Visualization in the Calculus. Mathematical Association of America Notes 19, 105–119.

[B46] Tall D. (1997). “Functions and calculus,” in International Handbook of Mathematics Education, ed A. J. Bishop (Dordrecht: Kluwer), 289–325.

[B47] Tall D. Vinner S. (1981). Concept image and concept definition in mathematics with particular reference to limits and continuity. Educ. Stud. Math. 12, 151–169. 10.1007/BF00305619

[B48] Tranel D. Kemmerer D. Adolphs R. Damasio H. Damasio A. R. (2003). Neural correlates of conceptual knowledge for actions. Cogn. Neuropsychol. 20, 409–432 10.1080/02643290244000248 20957578

[B49] Ulam S. (1958). John von Neumann 1903-1957. Bull. Amer. Math. Soc. 64, 1–49. 10.1090/S0002-9904-1958-10189-5

[B50] Varela F. J. Thompson E. Rosch E. (1991, 2016.) The Embodied Mind: Cognitive Science the Human Experience. Cambridge, MA: The MIT Press.

[B51] Vinner S. Dreyfus T. (1989). Images and definitions for the concept of function. J. Res. Math. Educ. 20, 356–366. 10.2307/749441

[B52] Wood S. J. Paloski W. H. Reschke M. F. (1998). Spatial coding of eye movements relative to perceived earth and head orientations during static roll tilt. Exp. Brain Res. 121, 51–58. 10.1007/s002210050436 9698190

[B53] Zhao M. Gersch T. M. Schnitzer B. S. Dosher B. Kowler E. (2012). Eye movements and attention: the role of pre-saccadic shifts of attention in perception, memory and the control of saccades. Vis. Res. 74, 40–60. 10.1016/j.visres.2012.06.017 22809798 PMC3623695

