# Supplementary material for: Sensorimotor Underpinnings of Mathematical Imagination: Qualitative Analysis
Source: Front Psychol. 2022 Jan 18;12:692602. doi: 10.3389/fpsyg.2021.692602 (PMC8803901; doi:10.3389/fpsyg.2021.692602)
Supplement: Supplementary file 1 [file Data_Sheet_1.PDF]

Instructions for Experiment on Orientation of Graphs and Their Components, Phase 1

We are interested in the imagery that arises when you imagine certain graphing tasks and perform certain visualizations while in various bodily positions. For each graphing task, we would appreciate it if you would perform it:
a) lying on your back,
b) lying on your right side,
c) lying on your left side,
d) face down (if that is comfortable to you, and we would appreciate a description of your head position), and
e) sitting or standing,
These positions may be taken in any sequence. Just please tell us your position in describing your experiences. However, please do each task in all positions before going on to the next task.

Before reading further, please choose and prepare a place where it will be convenient to lie in these positions and you won't be disturbed. For example, it could be a bed or a carpet on the floor. It should be comfortable for you, so that you are not distracted from the tasks.

Also, please prepare a way to take notes that is convenient to you and won't require you to disturb yourself much from the concentration of the visual imagery. For example, it could be paper and pencil, an audio recorder, or a computer nearby.

You can break the experiment into segments as you choose, taking notes at the end of each segment. In fact, we recommend starting with just one task -- or even the first task in one position. Just note for us how *you* engaged in the experiment, with the date and approximate time of day of each task and experience. Let us know how long you spend on each part of each task.

Play around with each task in turn! Please let us know what you have done and what happened. Provide as much detail -- text, illustrations, or both -- as possible.

Task 0. With your eyes closed, imagine the axes in a rectangular coordinate plane. Where are they? For example, is one axis parallel to your trunk? to your head if your head is not in line with your trunk? Is one axis vertical with respect to the earth? Where is the origin? Are the axes straight and orthogonal, and do they remain so?

Please perform the task in each position before going on to the next task. Please do that for each task.

Task 1. With your eyes closed, imagine the axes in a rectangular coordinate plane. Where are they? For example, is one axis parallel to your trunk? to your head if your head is not in line with your trunk? Is one axis vertical with respect to the earth? Where is the origin? Are the axes straight and orthogonal, and do they remain so?

On your axes, graph $y = x^2$. What does it look like? Where is the graph with respect to the coordinates? Does any movement occur? What is the orientation and movement, if any, with respect to the axes, with respect to the gravitational vertical, or with respect to your trunk or head?

Task 2. With your eyes closed, imagine the axes in a rectangular coordinate plane. Where are

they? For example, is one axis parallel to your trunk? to your head if your head is not in line with your trunk? Is one axis vertical with respect to the earth? Where is the origin? Are the axes straight and orthogonal, and do they remain so?

On your axes, graph $x = y^2$. What does it look like? Where is the graph with respect to the coordinates? Does any movement occur? What is the orientation and movement, if any, with respect to the axes, with respect to the gravitational vertical, or with respect to your trunk or head?

Task 3. With your eyes closed, imagine the axes in a rectangular coordinate plane. Where are they? For example, is one axis parallel to your trunk? to your head if your head is not in line with your trunk? Is one axis vertical with respect to the earth? Where is the origin? Are the axes straight and orthogonal, and do they remain so?

On your axes, graph $y = 1/x$. Imagine starting with negative x and moving along the function as x increases. Move gradually toward 0 and then positive x values.

What happens as you move along the graph of this function? Where is the graph with respect to the axes? Does any movement occur? What is the orientation and movement, if any, with respect the the axes, with respect to the earth, or with respect to your trunk or head?

Task 4. With your eyes closed, imagine the axes in a rectangular coordinate plane. Where are they? For example, is one axis parallel to your trunk? to your head if your head is not in line with your trunk? Is one axis vertical with respect to the earth? Where is the origin? Are the axes straight and orthogonal, and do they remain so?

On your axes, graph $y = x(x^2 - 1)$ and imagine starting with negative x and moving along the function as x increases. Move gradually toward 0 and then positive x values.

What happens as you move along the graph of this function? Where is the graph with respect to the axes? Does any movement occur? What is the orientation and movement, if any, with respect the the axes, with respect to the earth, or with respect to your trunk or head?

Task 5. With your eyes closed, imagine a cylinder. Describe it. Where is it? Is it parallel to your trunk? To your head? Is it vertical with respect to the earth?

Wrap a spiral around your cylinder, following the spiral as it wraps. What happens? What is your experience? How do the cylinder and spiral behave with respect to each other, to your trunk and head (or other body parts), or to the gravitational vertical?

Task 6. With your eyes closed, imagine a cone. Describe it. Where is it? Is it parallel to your trunk? To your head? Is it vertical with respect to the earth?

Wrap a spiral around a cone, following the spiral as it wraps. What happens? What is your experience? How do the cylinder and spiral behave with respect to each other, to your trunk and head (or other body parts), or to gravitational vertical?
